# Low-Valent Rhodium and Iridium Assemblies Directed by Uracilate and Guaninate Linkers

**DOI:** 10.1021/acs.inorgchem.6c01485

**Published:** 2026-07-06

**Authors:** Adrián Badía, Laura Asensio, M. Pilar del Río, José A. López, B. Eva Villarroya, Ana M. Geer, Cristina Tejel

**Affiliations:** Instituto de Síntesis Química y Catálisis Homogénea (ISQCH), Departamento de Química Inorgánica, 16379 CSIC−Universidad de Zaragoza, Pedro Cerbuna 12, Zaragoza 50009, Spain

## Abstract

Nucleobases combine rigid heterocyclic scaffolds with versatile N, O donor sets and intrinsic hydrogen-bonding capability, enabling access to diverse coordination architectures. Reactions of [{M­(μ-OMe)­(cod)}_2_] (M = Rh, Ir) with uracil (H_2_Ura) yield isostructural hexanuclear complexes [{M_2_(cod)_2_(μ_4_-κ^2^ *N* ^1^,*N* ^3^:κ^2^ *O* ^2^,*O* ^4^-Ura)}_3_] (M = Rh (**1**), Ir (**2**)) featuring a double-decker metallacalix[3]­arene motif sustained by dianionic μ_4_ uracilate linkers. In solution, **1** establishes an equilibrium with an octanuclear uracilate-based species [{Rh_2_(cod)_2_(Ura)}_4_] (**3**), as supported by DOSY NMR measurements. In contrast, reaction with guanine (H_2_Gua) affords the octanuclear complex [{Rh_2_(cod)_2_(μ_4_-κ^4^ *N* ^1^,*N* ^7^,*N* ^3^,*N* ^9^-Gua)}_4_] (**4**), in which each dianionic guaninate bridges four Rh­(I) centers through a μ_4_ coordination mode. The resulting Rh_8_ framework forms a closed metal–organic polyhedron with a gyrobifastigium topology reinforced by a continuous N–H···O hydrogen-bond belt. These results highlight how differences in nucleobase donor topology and hydrogen-bonding capability influence the assembly of low-valent Rh­(I) and Ir­(I) architectures.

## Graphical abstract

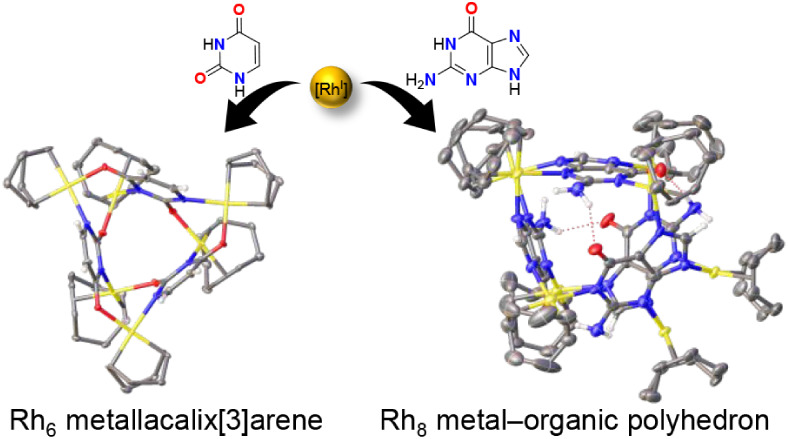

## Introduction

Discrete coordination architectures such as metallacalixarenes, metallacages, and metal–organic polyhedra (MOPs) bridge the fields of molecular and extended materials chemistry.^1–6^ Their well-defined geometries and modular connectivity have enabled applications in host–guest chemistry,^7,8^ gas storage,^9^ catalysis,^10^ and biomedicine.^11,12^ Despite their structural diversity, most reported examples rely on higher-valent metal centers or metal–metal-bonded secondary building units that impose rigid and predictable coordination geometries.^13,14^ Architectures sustained by low-valent, single-site metal nodes remain comparatively rare, particularly for square-planar d^8^ fragments such as Rh­(I) and Ir­(I), whose *cis* coordination geometry and absence of metal–metal bonding present distinct design challenges.

Within this landscape, MOPs are typically constructed from paddlewheel metal nodes, particularly Cu­(II) and Rh­(II), bridged by carboxylate ligands, giving rise to archetypal cuboctahedral and lantern-type structures.^3,4,6,15,16^ Such assemblies rely on metal–metal cores and predefined coordination vectors that favor predictable polyhedral topologies. While this strategy provides structural robustness, it limits access to architectures derived from single-site, low-valent square-planar metal fragments.

An alternative design approach involves linkers capable of multiple donor modes and secondary supramolecular interactions. Nucleobases offer such a platform, combining rigid heterocyclic scaffolds with diverse N, O donor sets and intrinsic hydrogen-bonding capability.^17^ Their defined donor positions and tautomeric flexibility make them attractive candidates for directing multinuclear assembly. In this work, we focus on uracil (H_2_Ura) and guanine (H_2_Gua), two heterocycles that present distinct donor topologies and hydrogen-bonding patterns (Figure 1).

**Figure 1:**
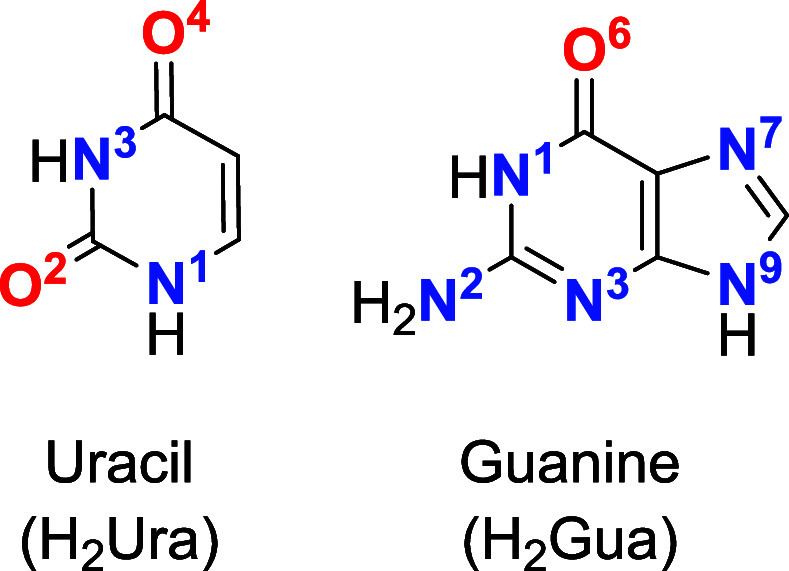
Structural representations and atom numbering scheme for uracil (H_2_Ura) and guanine (H_2_Gua).

Metal–nucleobase coordination chemistry displays rich structural diversity, from discrete dimers and metallacycles to multinuclear assemblies that emulate motifs of nucleic acids.^17,18^ Lippert, Sanz Miguel, and coworkers established platinum and palladium metallacalix­[n]­arenes bridged by uracil or cytosine, in which metal centers replace the methylene spacers of classical calixarenes.^19–25^ Related architectures based on Group 9 metals are far less common. Early Cp*Rh­(III) and Cp*Ir­(III) complexes with adenine or related ligands afforded triangular and hexanuclear motifs stabilized by π-stacking or μ-hydroxo bridges,^26–32^ while Suzuki and coworkers described Rh­(III)–thymine metallacalix[4]­arenes capable of cation encapsulation.^33,34^ Despite these advances, multinuclear assemblies sustained by nucleobases and low-valent Rh­(I) or Ir­(I) centers remain largely unexplored.

Here, we demonstrate that dianionic uracilate and guaninate ligands can direct the assembly of discrete multinuclear Rh­(I) and Ir­(I) architectures from square-planar {M­(cod)} (M = Rh, Ir) fragments. Depending on the nucleobase donor topology, the system forms either double-decker metallacalixarenes or an octanuclear metal–organic polyhedron with a gyrobifastigium topology, a relatively uncommon structural motif for discrete coordination cages and metal–organic polyhedra.

## Results and Discussion

### Uracil-Based Rhodium and Iridium Complexes

Reactions of [{M­(μ-OMe)­(cod)}_2_] (M = Rh, Ir) with uracil (H_2_Ura) afford a uracilate-directed μ_4_ assembly motif across low-valent Group 9 metals. In CH_2_Cl_2_/acetone (5:1), reaction with rhodium affords a yellow suspension, whereas the iridium analogue forms a red suspension. Under these conditions, the isostructural hexanuclear complexes [{M_2_(cod)_2_(μ_4_-κ^2^ *N* ^1^,*N* ^3^:κ^2^ *O* ^2^,*O* ^4^-Ura)}_3_] (M = Rh (**1**), Ir (**2**)) are obtained (Scheme 1).

**Scheme 1:**
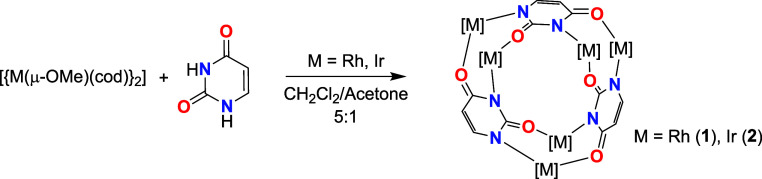
Reaction of [{M­(μ-OMe)­(cod)}_2_] (M = Rh, Ir) with Uracil in CH_2_Cl_2_/Acetone Yielding Complexes **1** and **2** ^a^

a [M] = M­(cod)

Slow diffusion of hexane into CH_2_Cl_2_ solutions afforded single-crystals of **1** and **2** suitable for X-ray diffraction. Both complexes adopt a double-decker metallacalix[3]­arene motif directed by μ_4_-uracilate linkers (Figures 2 and S19).^17^ The hexanuclear assemblies **1** and **2** consist of three {M_2_(cod)_2_} units interconnected by three doubly deprotonated uracilate ligands arranged in a head-to-tail orientation. Two distinct four-coordinate metal environments are present, defined by N1/O4 or N3/O2 donor sets from different uracilate ligands (Figure S20); in each case the metal center is additionally bound to two olefins from a cod ligand, adopting a distorted square-planar geometry.

**Figure 2:**
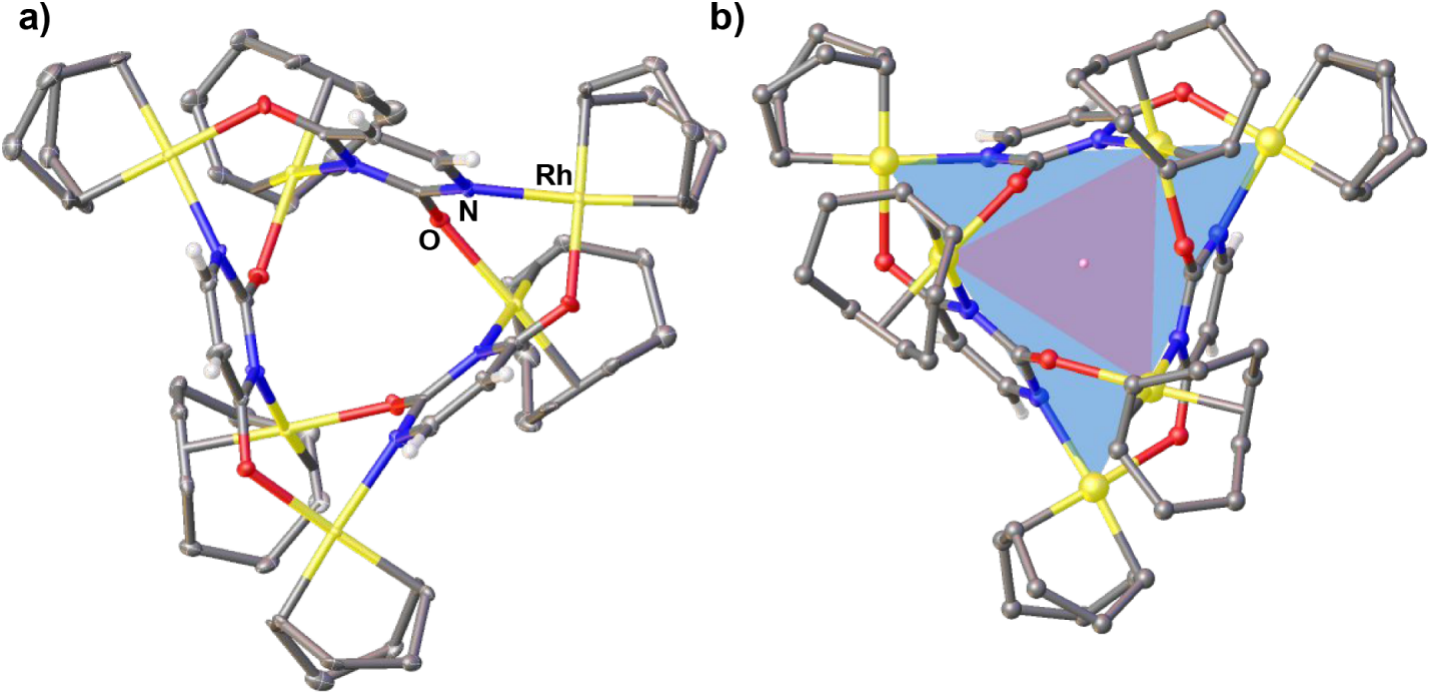
(a) Molecular structure of hexanuclear [{Rh_2_(cod)_2_(μ_4_-κ^2^ *N* ^1^,*N* ^3^:κ^2^ *O* ^2^,*O* ^4^-Ura)}_3_] (**1**). Hydrogen atoms, solvent molecules, and cod ligands are omitted for clarity. (b) Representation of **1** highlighting the two triangular Rh_3_ planes.

The six metal centers define two nearly planar M_3_ triangular decks related by approximate *C*_3_ symmetry. In **1**, the Rh_3_ planes are separated by 2.3274(5) Å and rotated by 25.07° about the pseudo-*C*_3_ axis, whereas in **2** the corresponding Ir_3_ planes are slightly closer (2.2398(2) Å) with a smaller relative rotation of 23.94°. In both cases, intradeck M···M separations exceed typical bonding distances, consistent with the absence of direct metal–metal interactions. The lower deck spans a larger triangular area than the upper deck, giving rise to an asymmetric double-decker arrangement.^35^

The metal–nitrogen and metal–oxygen bond distances in both complexes fall within the range reported for metal–nucleobase systems,^30,33^ while intramolecular metal···metal contacts remain nonbonding, with slightly shorter contacts observed for the iridium derivative. Bond metrics within the uracilate ligands in both structures support a doubly deprotonated formulation consistent with the 2,4-dioxo tautomer observed in related systems.^17^ Subtle intraligand C–N bond asymmetry in **2**, relative to **1**, suggests a greater degree of π localization in the iridium complex.^36^

The ^1^H NMR spectrum of **1** in CDCl_3_ is consistent with its *C*_3_ symmetry, showing two doublets for the uracilate protons and two distinct sets of cod resonances corresponding to the two Rh environments (Figure 3a). These data indicate the equivalence of the three {Rh_2_(cod)_2_(Ura)} fragments and are consistent with a symmetric multinuclear assembly.

**Figure 3:**
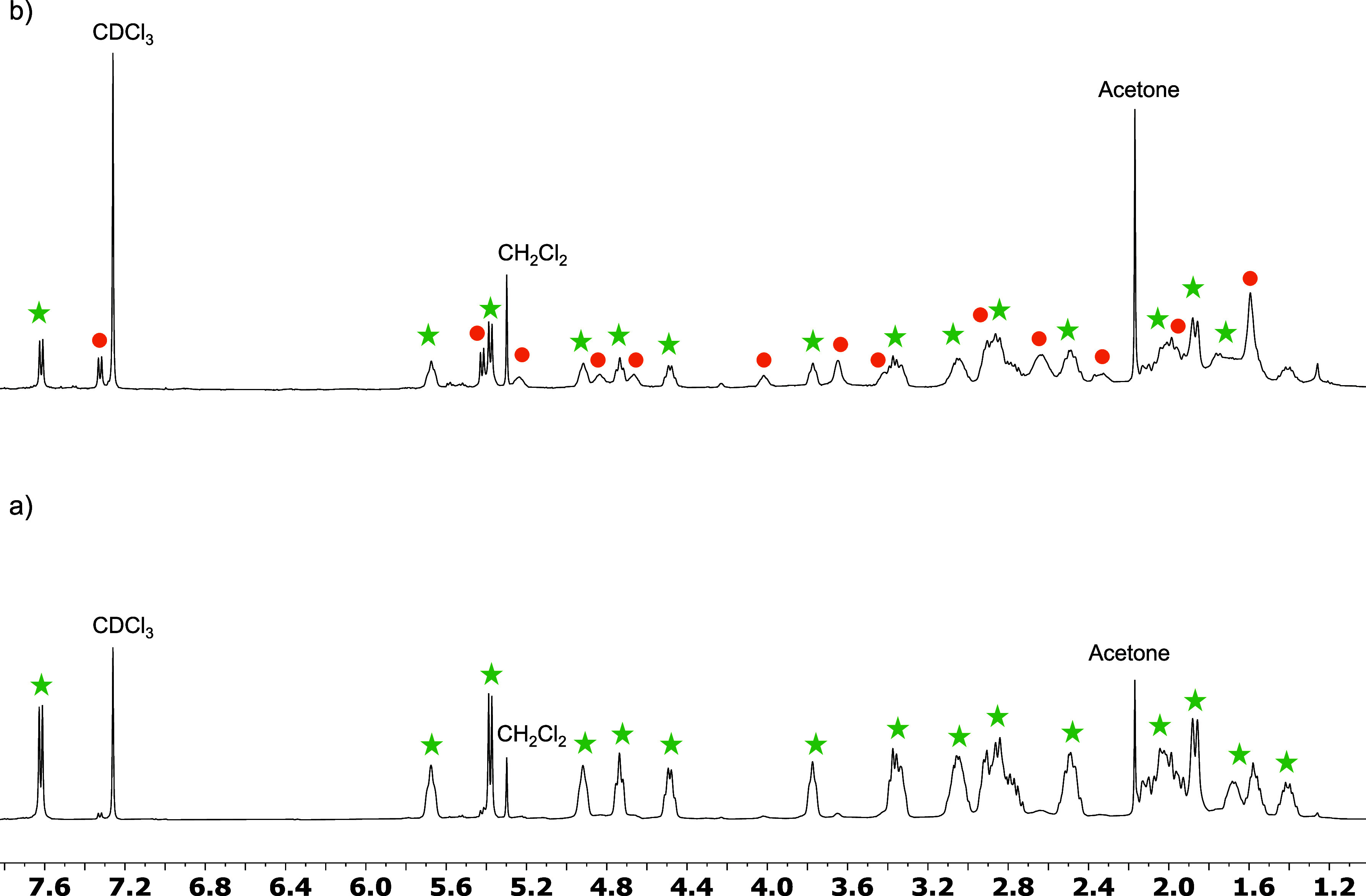
(a) ^1^H NMR spectrum of [{Rh_2_(cod)_2_(μ_4_-κ^2^ *N* ^1^,*N* ^3^:κ^2^ *O* ^2^,*O* ^4^-Ura)}_3_] (**1**) in CDCl_3_, after 10 min at 25 °C. (b) Spectrum after 90 min, showing the conversion of **1** toward the equilibrium mixture of **1** and **3**. Signals for **1** are marked with green stars and those for **3** with orange circles.

In solution at 25 °C, **1** establishes an equilibrium with a second species [{Rh_2_(cod)_2_(Ura)}_4_] (**3**), whose relative proportion is influenced by both temperature and the reaction medium (Figures 3 and S1). Preparations performed in acetone afford samples enriched in **3**, whereas reactions performed in CH_2_Cl_2_/acetone mixtures preferentially yield **1**. Complex **3** was isolated from acetone as a yellow solid but did not yield single-crystals suitable for diffraction. The ^1^H NMR spectrum of **3** in C_6_D_6_ shows two doublets for the uracilate protons and two sets of olefinic signals for the cod ligands, indicating a symmetry pattern analogous to that observed for **1** in solution (Figure S7).

Although single-crystals of **3** suitable for X-ray diffraction could not be obtained, its formulation as an octanuclear species is strongly supported by DOSY NMR measurements. Diffusion coefficients of 6.35 × 10^–10^ and 5.06 × 10^–10^ m^2^ s^–1^ were determined for **1** and **3**, corresponding to hydrodynamic radii (*r*_H_) of 6.74 and 8.46 Å, respectively. The r_H_ value measured for **1** closely matches that estimated from its crystallographically determined dimensions, supporting retention of the compact hexanuclear assembly in solution. In contrast, the larger hydrodynamic radius of **3** is consistent with that expected for the octanuclear Rh­(I) species [{Rh_2_(cod)_2_(Ura)}_4_] (Figure S16).

The two complexes were found to be in equilibrium, with the distribution shifting toward **3** at 50 °C, although **1** remained the major species over the temperature range examined (Figure S1). Below 30 °C, equilibration became considerably slower, preventing reliable measurements on experimentally accessible time scales.

Cyclic voltammetry of **1** in CH_2_Cl_2_ displayed three quasi-reversible one-electron oxidation processes at *E*_1/2_ = 0.03, 0.28, and 0.50 V vs Fc^+/0^ (Figure 4), assigned to the metal-centered Rh­(I) to Rh­(II) oxidations. The oxidation events occur sequentially with substantial separation between consecutive redox couples (ΔE = 210–250 mV), indicating that the rhodium centers do not behave as electrochemically independent sites. Despite the presence of only two crystallographically distinct rhodium environments, three different redox couples are observed, consistent with cooperative redox processes within the compact Rh_6_ assembly and suggesting moderate electronic communication between the metal centers.^37,38^

**Figure 4:**
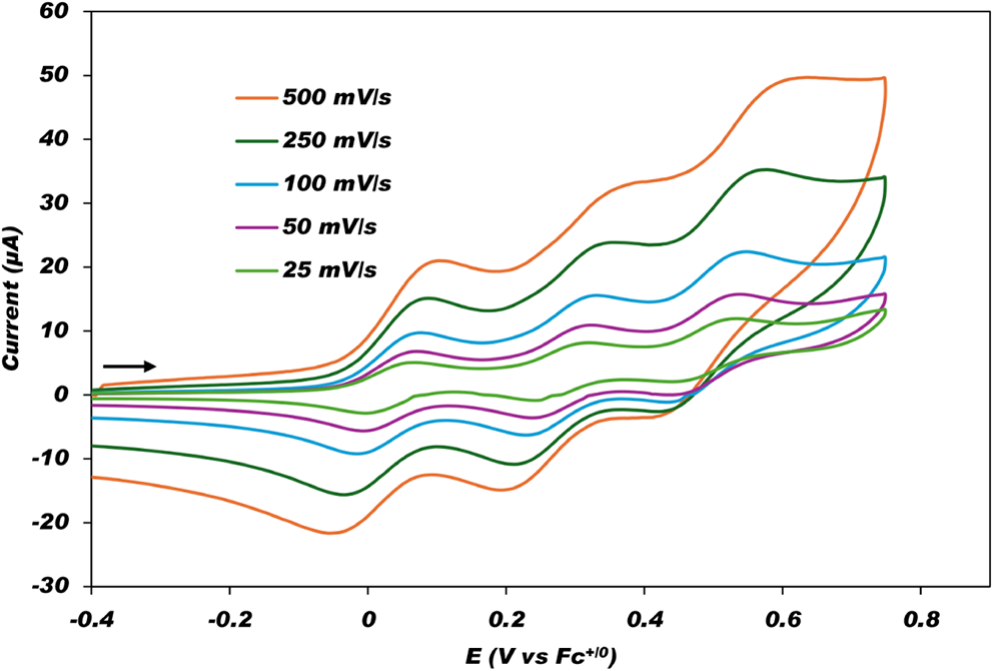
Cyclic voltammogram of **1** (1.0 mM) in CH_2_Cl_2_ with 0.1 M [NBu_4_]­[PF_6_] as a supporting electrolyte recorded at variable scan rates (25 mV/s to 500 mV/s).

The iridium analogue **2** is markedly less soluble than **1** in common organic solvents. The ^1^H NMR spectrum in CD_2_Cl_2_ shows two doublets for the uracilate protons and broad cod resonances (Figure S8), consistent with an overall *C*_3_-symmetric environment and chemical equivalence of the three {Ir_2_(cod)_2_(Ura)} fragments in solution.

### Guanine-Based Rhodium Complexes

In contrast to uracil, whose N, O donor set favors layered assemblies, guanine introduces an expanded nitrogen-rich coordination environment capable of redirecting the assembly pathway.^17,18^ Most reported guanine complexes involve neutral or monoanionic coordination through N7, with O6 and the exocyclic amino group engaged in hydrogen bonding. Multinuclear assemblies remain uncommon, and examples involving doubly deprotonated guanine are particularly rare.^39^

Initial attempts to reproduce the uracil chemistry using [{Rh­(μ-OMe)­(cod)}_2_] as a rhodium precursor proved unsuccessful. In DMF, where both reagents dissolved, IR spectroscopy indicated formation of Rh–CO species derived from solvent carbonylation. Successful coordination was achieved instead by first deprotonating guanine with KOH in methanol, followed by addition of [{Rh­(μ-Cl)­(cod)}_2_], which produced a yellow suspension that, after workup, affords the octanuclear [{Rh_2_(cod)_2_(μ_4_-κ^4^ *N* ^1^,*N* ^7^,*N* ^3^,*N* ^9^-Gua)}_4_] (**4**) as a yellow solid (Scheme 2). Attempts to prepare the corresponding iridium analogue under analogous conditions did not afford isolable products.

**Scheme 2:**
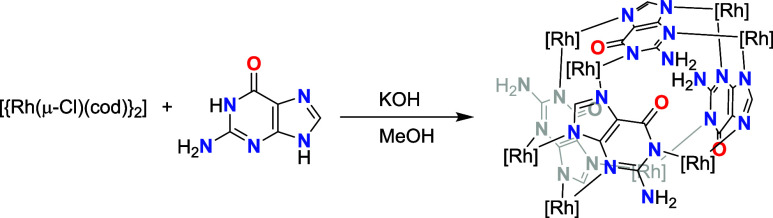
Reaction of [{Rh­(μ-Cl)­(cod)}_2_] with Guanine and KOH in MeOH Yielding Complex **4** [Fn sch2-fn2]

a [Rh] = Rh­(cod)

Single-crystal X-ray diffraction of **4** reveals that the change in donor topology does not simply modify the layered motif observed for uracil, but instead redirects the system toward a closed metal–organic polyhedron (MOP) in which eight Rh­(I)­(cod) units are interconnected by four μ_4_-guaninate ligands (Figure 5a). Each Rh atom adopts a square-planar geometry defined by two olefins from cod and two nitrogen donors from different guaninate ligands. Two distinct Rh environments are observed: one type bound by N1/N7, and the other by N3/N9 (Figure S22). In this rare binding pattern for guanine, each dianionic guaninate links four Rh­(I) centers, enforcing a head-to-tail orientation in which the carbonyl groups are rotated by approximately 180°. The imidazole bond pattern indicates enhanced C8 = N7 character, consistent with a keto–N7 resonance form of the guaninate dianion.

**Figure 5:**
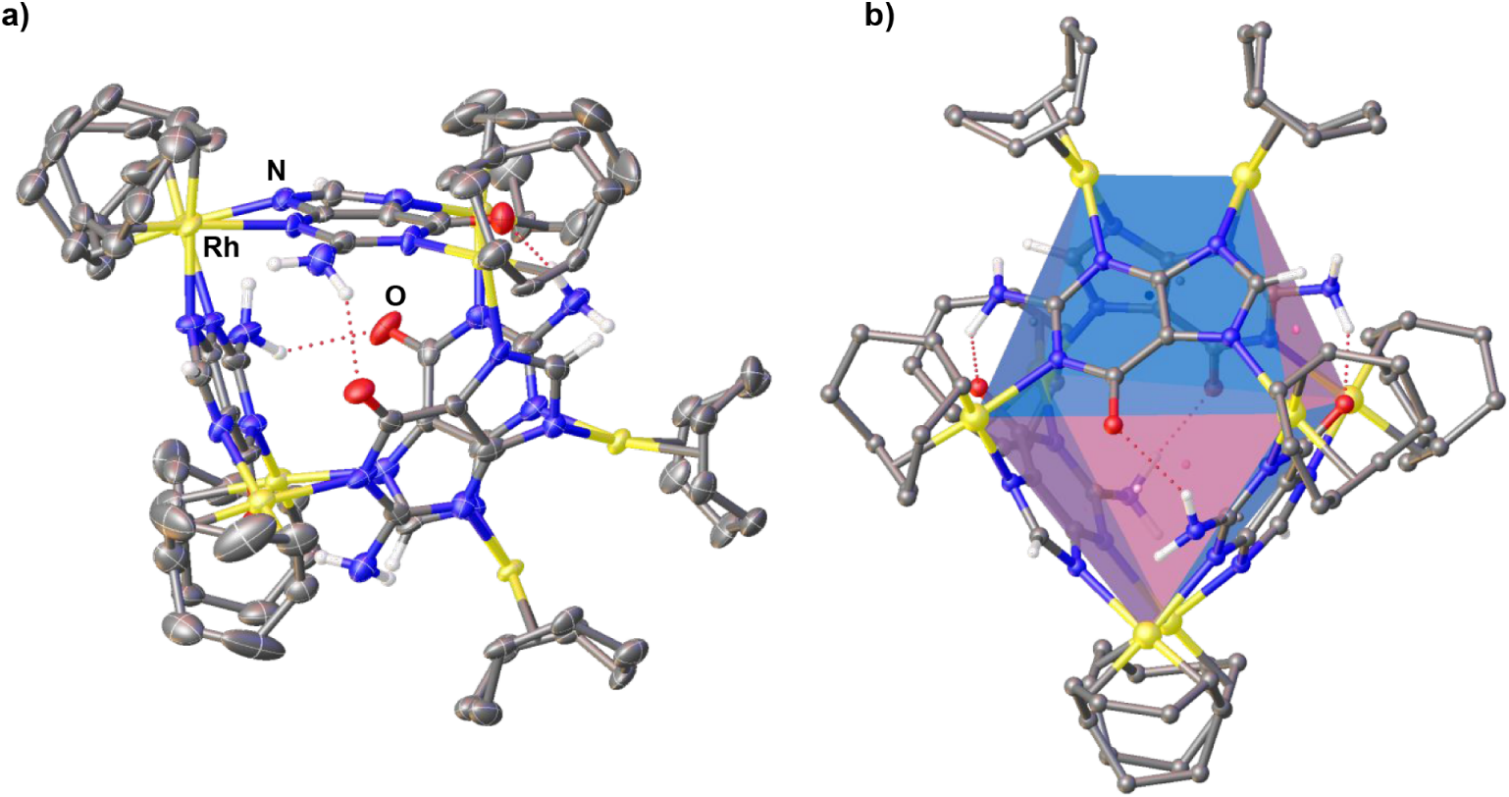
(a) Molecular structure of [{Rh_2_(cod)_2_(μ_4_-κ^4^ *N* ^1^,*N* ^7^,*N* ^3^,*N* ^9^-Gua)}_4_] (**4**). (b) Representation of **4** illustrating the gyrobifastigium topology, with triangular and trapezoidal faces highlighted. Hydrogen atoms from the cod ligand and solvent molecules are omitted for clarity.

The Rh_8_ framework conforms to a gyrobifastigium (GBF) topology (Figure 5b), a Johnson polyhedron characterized by eight vertices and 14 edges grouped in an A_4_B_4_ arrangement. The resulting cage comprises four triangular and four trapezoidal faces joined in a staggered configuration, placing **4** among the few molecular GBF polyhedra reported in coordination chemistry.^40^ Although gyrobifastigium geometries have occasionally been identified in metal clusters and polyoxometalates, they remain rare in coordination cages and metal–organic polyhedra. The emergence of this topology from square-planar Rh­(I) nodes and flexible nucleobase linkers highlights a connectivity pattern distinct from classical cuboctahedral MOPs derived from paddlewheel nodes. The square-planar coordination geometry of the Rh­(I) centers enforces approximately orthogonal binding vectors, while the guaninate ligands bridge four metal centers in a μ_4_ fashion, collectively favoring the staggered triangular–trapezoidal arrangement required for the gyrobifastigium polyhedron.

In contrast to Rh-based MOPs constructed from Rh_2_ paddlewheel clusters and rigid dicarboxylate linkers,^41–43^ where the Rh–Rh bonded unit acts as a predefined 4-connected node directing cuboctahedral assemblies, the Rh_8_–GBF cage is built from mononuclear square-planar Rh­(I) vertices without metal–metal interactions. Its topology arises from μ_4_-dianionic guaninate bridges and is further stabilized by a directional hydrogen-bond belt, representing a distinct Rh-based polyhedral motif that does not rely on classical paddlewheel-driven design.

Beyond metal coordination, the guaninate ligands engage in a cooperative hydrogen-bonding network that reinforces the Rh_8_ framework. Each exocyclic −NH_2_ donor interacts with the O6 carbonyl of a neighboring ligand, while its own O6 accepts a hydrogen bond from another −NH_2_ group, generating a continuous N–H···O belt around the cage. The hydrogen bonds display H···O distances of 1.87–2.14 Å and donor–acceptor separations of 2.80–2.85 Å, with one nearly linear contact (N10–H10B···O4, 170.5°), consistent with moderate to strong interactions (Table S2). This supramolecular reinforcement is reminiscent of guanine quartets.^44^ Metal–nucleobase chemistry has been inspired both by metal-modified base pairs,^18,45^ where coordination replaces or complements hydrogen bonding in canonical base pairs, and by guanine quartets stabilized by Hoogsteen interactions and alkali cations.^46^ Complex **4** integrates both paradigms: guanine binds four Rh­(I) centers through a previously unreported μ_4_ coordination mode while sustaining a cooperative hydrogen-bond belt, combining metal coordination and base-pairing principles within a discrete multinuclear cage. Consistent with this closed architecture, inspection of the molecular model shows that **4** contains a small internal cavity capable of accommodating an approximately 5 Å diameter sphere (Figure S23).

In solution, complex **4** appears as a single species by ^1^H NMR spectroscopy in CDCl_3_, showing two singlets in the aromatic region (δ 7.83 for H^8^, 7.71 for NH_2_) and eight olefinic resonances between δ 3.4–4.8, consistent with two types of cod ligands and the [{Rh_2_(cod)_2_(μ-Gua)}_4_] formulation (Figure 6a). Further support for the structural model of **4** in solution comes from ^1^H,^1^H-NOESY spectroscopy (Figure S11). NOESY crosspeaks between guanine H^8^ and olefinic resonances from both cod ligands, together with the observed NMR symmetry, indicate that each guaninate is positioned between two {Rh_2_(cod)_2_} nodes in solution, consistent with the μ_4_ topology.

**Figure 6:**
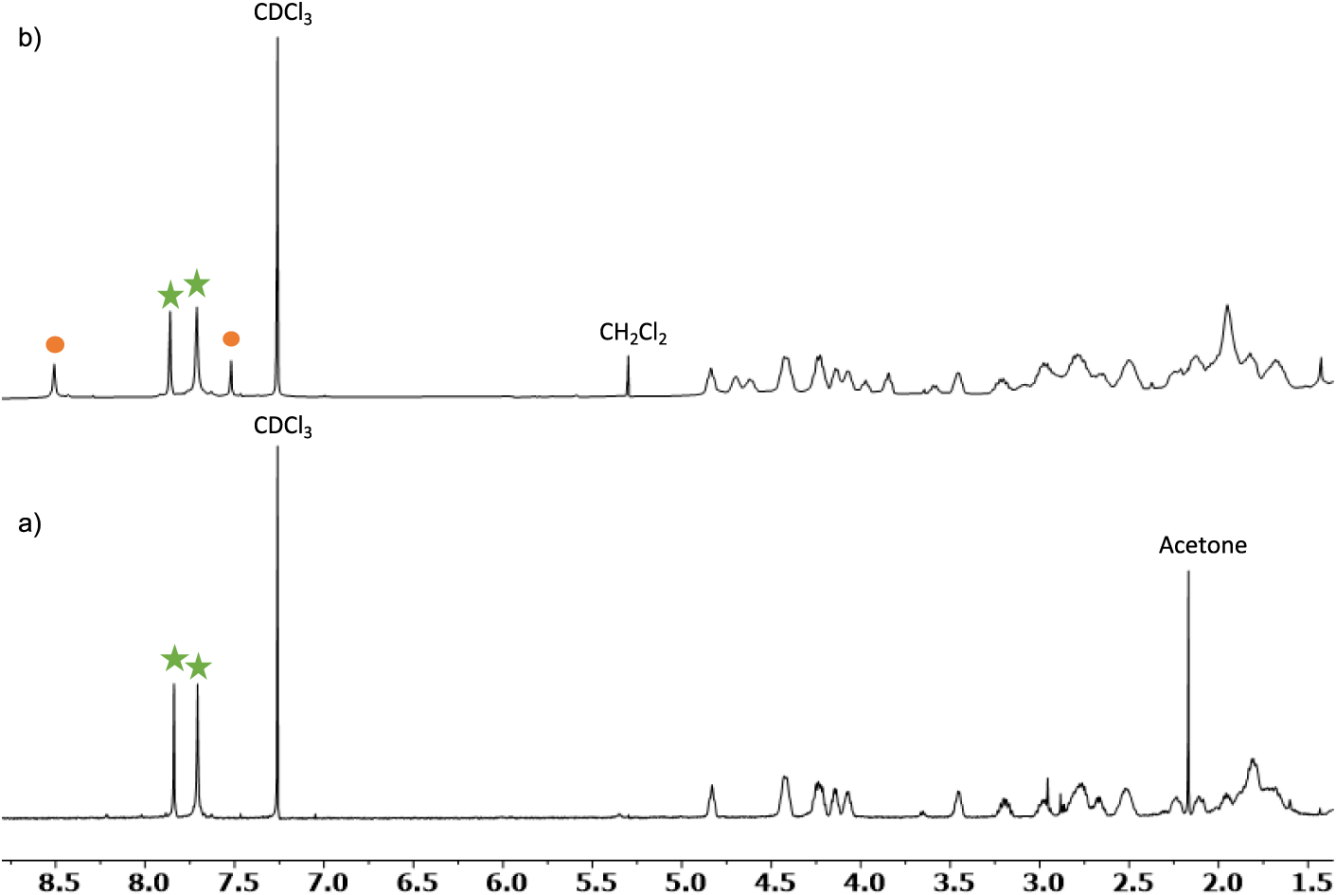
(a) ^1^H NMR spectrum of [{Rh_2_(cod)_2_(μ_4_-κ^4^ *N* ^1^,*N* ^7^,*N* ^3^,*N* ^9^-Gua)}_4_] (**4**) in CDCl_3_ at 25 °C. (b) ^1^H NMR spectrum of a mixture of complex **4** and **5** at 25 °C. Guanine signals for **4** are marked with green stars and those for **5** with orange circles.

In repeated preparations performed under inert conditions, a second guanine-based species [{Rh_2_(cod)_2_(Gua)}_4_] (**5**) was detected together with **4**. The ^1^H NMR spectrum of **5** displays a closely related symmetry pattern to that of **4**, albeit with distinct chemical shifts for both guanine and cod resonances (Figure 6b). The relative ratio of **5** and **4** depends on the time allowed before isolation: immediate workup after reflux gave an approximate 45:55 mixture of **5** and **4**, whereas stirring at room temperature for 16 h before workup shifted the ratio to approximately 13:87 (Figure S14). Accordingly, NMR monitoring showed that **5** gradually evolves into **4** in solution (Figure S15). These observations support the assignment of **5** as a metastable guaninate based Rh­(I) assembly that evolves toward the thermodynamically favored cage **4** under the conditions studied.

Although **5** could not be isolated in analytically pure form, DOSY NMR measurements of **4** and **5** in CDCl_3_ gave nearly identical diffusion coefficients (4.72 × 10^–10^ and 4.64 × 10^–10^ m^2^ s^–1^), corresponding to hydrodynamic radii of 9.22 and 9.38 Å, respectively, consistent with species of comparable size. Taken together, the similar NMR symmetry, comparable hydrodynamic dimensions, and clean spectroscopic evolution from **5** to **4** support assignment of **5** as a closely related guanine based Rh­(I) assembly, likely corresponding to an isomeric octanuclear Rh­(I) framework.

Cyclic voltammetry measurements of **4** reveal a more complex electrochemical response than that observed for **1**, with multiple broad and partially overlapping anodic features at E_p,a_ = 0.16, 0.28, 0.45, 0.72, and 0.91 V vs Fc^^+^/0^ at 25 mV/s (Figures S17 and S18). The presence of several distinct oxidation features suggests that the Rh centers do not behave as independent redox sites, consistent with redox communication within the guanine-directed Rh_8_ cage. However, the broadness and irreversibility of these processes preclude a more detailed assignment.

## Conclusions

This work demonstrates that nucleobases can act as versatile dianionic linkers for constructing multinuclear Rh­(I) and Ir­(I) architectures that bridge metallacalixarene and MOP chemistry. Uracil promotes the formation of double-decker metallacalix[3]­arenes, an unusual motif in metallacalixarene chemistry, and demonstrates how subtle changes in reaction conditions can shift the balance between compact and more expanded uracilate-based assemblies. In contrast, guanine affords an octanuclear Rh­(I) MOP featuring a previously unobserved μ_4_ guaninate binding mode and a gyrobifastigium topology reinforced by a cooperative hydrogen-bond belt. Electrochemical studies of the Rh­(I) assemblies reveal nonindependent redox behavior within these multinuclear frameworks. The separated quasi-reversible oxidations observed for **1** and the multiple separated anodic features displayed by **4** indicate that the Rh centers do not behave as equivalent, independent redox sites, supporting redox communication within the nucleobase-directed architectures.

Although both uracil and guanine act as tetrapodal dianionic linkers, their distinct donor distributions lead to fundamentally different architectural outcomes. In uracil, the μ_4_ coordination involves both carbonyl groups and N1/N3 donors, yielding rigid pillars that stabilize open, layered double-decker architectures. Guanine, in contrast, binds solely through nitrogen donors, leaving its carbonyl and amino groups available for secondary N–H···O interactions that close the framework into a polyhedral cage. Subtle intraligand differences between Rh and Ir complexes further suggest enhanced π-delocalization in the Rh system relative to its Ir analogue.

Unlike coordination cages commonly assembled from neutral ditopic ligands with predefined coordination vectors, the present frameworks arise from the interplay between single-site square-planar Rh­(I)/Ir­(I) metal nodes and dianionic nucleobases capable of μ4 coordination. The metal centers impose a cis geometry characteristic of d8 square-planar nodes, while the nucleobase linkers dictate overall connectivity through adaptable N, O donor arrangements and secondary hydrogen-bonding interactions. The resulting architectures are therefore not programmed by metal–metal bonded secondary building units or predesigned angular clips, but instead emerge from geometric compatibility between low-valent square-planar nodes and heterocyclic linkers. This study further demonstrates that nucleobase ligands can serve as versatile multitopic connectors capable of directing the assembly of low-valent transition-metal architectures with unusual topologies.

## Experimental Section

### General Methods

All of the operations were performed under an argon atmosphere using standard Schlenk techniques. Organic solvents were dried by standard procedures and distilled under argon prior to use or obtained oxygen- and water-free from a Solvent Purification System. All reagents and solvents were purchased from commercial sources and used without further purification unless stated otherwise. Complexes [{Rh­(μ-Cl)­(cod)}_2_],^47^ [{Ir­(μ-Cl)­(cod)}_2_],^48^ and [{Rh­(μ-OMe)­(cod)}_2_][Bibr ref49] were prepared according to literature procedures. Carbon, hydrogen, and nitrogen analyses were performed on vacuum-dried bulk samples, with a PerkinElmer 2400 CHNS/O microanalyzer. In cases where residual solvent molecules were included in the analytical formulas, their presence was verified by ^1^H NMR spectroscopy. High resolution ESI mass spectra were recorded from acetonitrile solutions on a Bruker MicroTof-Q instrument equipped with an electrospray ionization source and a hybrid quadrupole time-of-flight analyzer (Q TOF). MALDI-TOF mass spectra were acquired on a Bruker Microflex mass spectrometer (low resolution) or on a Bruker TIMS-TOF-FLEX instrument (high resolution) using DCTB (*trans*-2-[3-(4-*tert*-butylphenyl)-2-methyl-2-propenylidene]­malononitrile) as the matrix. IR spectra of solid samples were recorded with a PerkinElmer 100 FT-IR spectrometer (4000–400 cm^–1^) equipped with attenuated total reflectance (ATR). NMR spectra were recorded on Bruker AV300, AV400 and AV500 spectrometers operating at 300.13, 400.13, and 500.13 MHz, respectively, for ^1^H. Chemical shifts are quoted in ppm relative to SiMe_4_, using the internal signal of the deuterated solvent (for ^1^H and ^13^C). DOSY experiments were performed using the PFGSE (Pulsed-Field Gradient Spin–Echo) NMR diffusion method and analyzed with the Bruker software on an NMR AV400 spectrometer. All DOSY spectra were acquired spinning at 30 °C. The variation of the intensity of a selected signal in the ^1^H NMR spectra (*I*) as a function of the gradient strength (G) is described by the equation: Ln­(*I*/*I* ^0^) = – γ^2^δ^2^Gi^2^(Δ−δ/3)*D*, where γ = gyromagnetic ratio of the proton, δ = length of the gradient pulse, Gi = gradient strength, Δ = delay between the midpoints of the gradients, and *D* = diffusion coefficient.^50^ Before recording the DOSY experiment, the values of δ (small delta) and Δ (big delta) were optimized for each complex by using the 1D pulse sequence for diffusion measurements (ledbpgp2s1d, δ (2 × P30) and Δ (d20), Bruker software). The selected values provided a considerable reduction of the intensity of the signal, while remaining sufficient signal for integration. Next, the 2D DOSY experiment (ledbpgp2s pulse sequence) was recorded using the optimized δ and Δ*v*alues, with the G incremented over 16 steps. Data were analyzed with the Bruker software, which directly provided the diffusion coefficient (*D*). The quality of the data was tested by representation Ln­(*I*/*I* ^0^) versus G2, which gave an excellent fit to a straight line in all the cases. Hydrodynamic radii (*r*_H_) were calculated from the Stokes–Einstein equation: $r_{H}=\frac{kT}{6\pi \eta D}$ (where T is absolute temperature, Κ is the Boltzmann constant, η is the solvent viscosity and *D* is the diffusion coefficient).
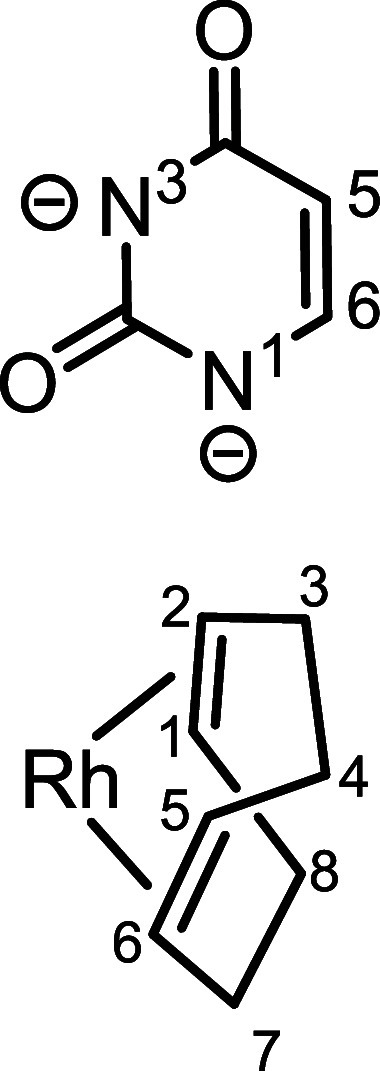


#### Synthesis of [{Rh_2_(cod)_2_(μ_4_-κ^2^ *N^1^*,*N* ^3^:κ^2^ *O^2^*,*O* ^4^-Ura)}_3_] (**1**)

Uracil (25.0 mg, 0.22 mmol) was suspended in 10 mL of acetone and refluxed for 15 min to improve its solubility. After cooling to room temperature, [{Rh­(μ-OMe)­(cod)}_2_] (108.0 mg, 0.22 mmol) was added, yielding a yellow suspension to which 3 mL of CH_2_Cl_2_ were then added. The mixture was stirred at room temperature for 24 h. The yellow solid was collected by filtration, washed with acetone at 0 °C (3 × 2 mL), and vacuum-dried. Yield: 90.9 mg (78%). Single-crystals suitable for X-ray diffraction were obtained by slow diffusion of hexane into a CH_2_Cl_2_ solution at 5 °C. IR v/cm^–1^ (ATR): 1716 (w), 1582 (s), 1529 (s), 1460 (s), 1429 (s). ^1^H NMR (500 MHz, CDCl_3_, 25 °C): δ = 7.64 (d, *J* = 6.5 Hz, 3H, H^5^–Ura), 5.74 (br t, 3H, H^1^-cod^A^), 5.31 (d, *J* = 6.5 Hz, 3H, H^6^–Ura), 4.97 (m, 3H, H^2^-cod^A^), 4.75 (t, 3H, H^1^-cod^B^), 4.48 (m, 3H, H^2^-cod^B^), 3.77 (br t, 3H, H^5^-cod^A^), 3.47 (t, *J* = 6.9 Hz, 3H, H^5^-cod^B^), 3.33 (br t, 3H, H^6^-cod^A^), 3.09 (m, 3H, H^8a^-cod^B^), 3.05 (m, 3H, H^8a^-cod^A^), 3.00 (m, 3H, H^6^-cod^B^), 2.89 (m, 3H, H^4a^-cod^A^), 2.86 (m, 3H, H^3a^-cod^A^), 2.78 (m, 3H, H^8a^-cod^B^), 2.47 (m, 3H, H^3a^-cod^B^), 2.51 (m, 3H, H^7a^-cod^A^), 2.13 (m, 3H, H^7a^-cod^B^), 2.03 (m, 6H, H^8b^-cod^A^ + H^8b^-cod^B^), 1.98 (m, 3H, H^2b^-cod^B^), 1.89 (m, 3H, H^4b^-cod^A^), 1.86 (m, 3H, H^3b^-cod^A^), 1.82 (m, 3H, H^7b^-cod^A^), 1.58 (m, 3H, H^3b^-cod^B^), 1.44 (m, 3H, H^7b^-cod^B^). ^13^C­{^1^H} (126 MHz, CDCl_3_, 25 °C): 179.1 (OC4-Ura), 168.1 (OC^2^-Ura), 155.6 (C^6^–Ura), 102.1 (C^5^–Ura), 89.6 (d, C^1^-cod^B^), 85.5 (d, C^1^-cod^A^), 78.3 (d, C^2^-cod^A^), 76.1 (d, C^2^-cod^B^), 74.2 (d, C^6^-cod^B^), 73.4 (d, C^6^-cod^A^), 71.7 (d, C^5^-cod^A^), 70.1 (d, C^5^-cod^B^), 33.6 (C^4^-cod^B^), 33.0 (C^3^-cod^A^), 32.4 (C^8^-cod^B^), 31.1 (C^7^-cod^A^), 30.7 (C^8^-cod^A^), 30.3 (C^4^-cod^A^), 29.9 (C^7^-cod^B^), 28.9 (C^3^-cod^B^). HR-ESI^+^-MS: *m*/*z* calcd for [Rh_6_C_60_H_78_N_6_O_6_] + Na [M + Na]^+^ 1619.0205, found: 1619.0236 (err [mDa] = 3.1). Anal. Calcd for C_60_H_78_O_6_N_6_Rh_6_: C 45.13 H 4.92, N 5.26; found: C 45.26, H 5.03, N 5.15.

#### Synthesis of [{Ir_2_(cod)_2_(μ_4_-κ^2^ *N^1^*,*N* ^3^:κ^2^ *O^2^*,*O* ^4^-Ura)}_3_] (**2**)

Uracil (17.1 mg, 0.15 mmol) was suspended in 10 mL of acetone and refluxed for 15 min to improve its solubility. After cooling to room temperature, [{Ir­(μ-OMe)­(cod)}_2_] (101.1 mg, 0.15 mmol) was added. The mixture was stirred at room temperature for 2 h, during which the initial yellow suspension gradually turned red. The resulting red solid was separated by filtration, washed with acetone (3 × 2 mL), and vacuum-dried. Single-crystals suitable for X-ray diffraction were obtained by slow diffusion of hexane into a CH_2_Cl_2_ solution at room temperature. Yield: 89.8 mg (84%). IR v/cm^–1^ (ATR): 1766 (w), 1714 (w), 1642 (w), 1586 (s), 1451 (s), 1417 (s), 1390 (s). Selected ^1^H NMR (500 MHz, CD_2_Cl_2_, 25 °C) signals: δ = 7.63 (d, *J* = 6.6 Hz, 3H, H^5^–Ura), 5.54 (d, *J* = 6.6 Hz, 3H, H^6^–Ura), 4.6 (t, *J* = 7.0 Hz, 1H, cod), 4.4 (s, 2H), 4.0 (m, 1H), 3.5 (d, *J* = 7.4 Hz, 1H), 3.2 (t, *J* = 6.8 Hz, 1H). MS (MALDI-TOF^+^): *m*/*z* (%) = 2134.4 (5) [Ir_6_(C_8_H_12_)_6_(C_4_H_2_N_2_O_2_)_3_^+^]; 1422.3 (100) [Ir_4_(C_8_H_12_)_4_(C_4_H_2_N_2_O_2_)_2_^+^]. Anal. Calcd for C_60_H_78_O_6_N_6_Ir_6_·2CH_2_Cl_2_: C 32.34 H 3.59, N 3.65; found: C 32.47, H 3.82, N 3.77.
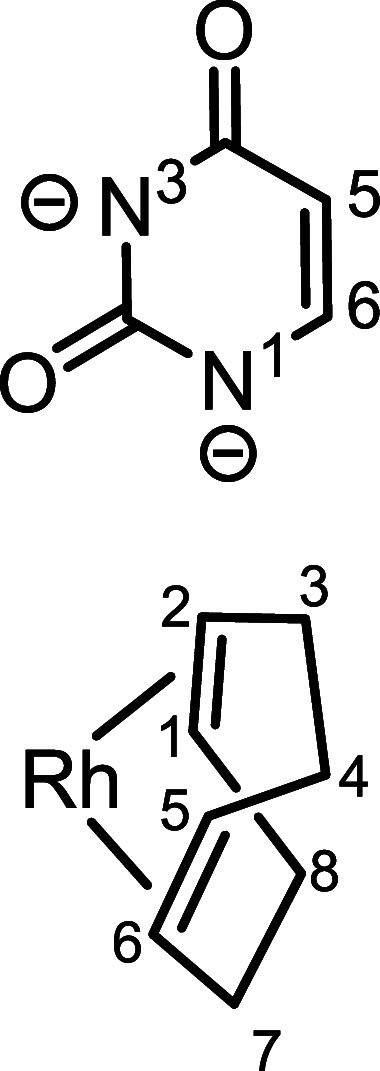


#### Synthesis of [{Rh_2_(cod)_2_(Ura)}_4_] (**3**)

Uracil (24.3 mg, 0.22 mmol) was suspended in 10 mL of acetone and refluxed for 15 min to improve its solubility. After cooling to room temperature, [{Rh­(μ-OMe)­(cod)}_2_] (105.0 mg, 0.22 mmol) was added, yielding a yellow suspension. The mixture was stirred at room temperature for 24 h, after which the resulting yellow solid was collected by filtration, washed with acetone (3 × 2 mL), and vacuum-dried. Yield: 90.1 mg (77%). ^1^H NMR (500 MHz, C_6_D_6_, 25 °C): δ = 7.14 (d, *J* = 6.5 Hz, 2H, H^5^–Ura), 5.52 (d, *J* = 6.5 Hz, 2H, H^6^–Ura), 5.74 (m, 2H, H^1^-cod^A^), 5.62 (m, 2H, H^2^-cod^A^), 5.16 (m, 2H, H^1^-cod^B^), 5.06 (br t, 2H, H^2^-cod^B^), 4.24 (br, 2H, H^6^-cod^B^), 3.98 (m, 4H, H^5+6^-cod^A^), 3.42 (m, 2H, H^5^-cod^B^), 2.89 (m, 2H, H^3A^-cod^A^), 2.92 (m, 2H, H^7a^-cod^B^), 2.66 (m, 2H, H^4A^-cod^A^), 2.63 (m, 2H, H^7A^-cod^A^), 2.58 (m, 2H, H^8a^-cod^B^), 2.52 (m, 2H, H^3a^-cod^B^), 1.94 (m, 2H, H^8A^-cod^A^), 1.88 (m, 2H, H^3B^-cod^A^), 1.76 (m, 2H, H^7b^-cod^B^), 1.73 (m, 2H, H^3b^-cod^B^), 1.67 (m, 2H, H^7B^-cod^A^), 1.65 (m, 2H, H^4B^-cod^A^), 1.56 (m, 2H, H^8b^-cod^B^), 1.36 (m, 2H, H^4b^-cod^B^). ^13^C­{^1^H} (126 MHz, CDCl_3_, 25 °C): δ = 182.2 (OC-Ura), 172.2 (OC-Ura), 154.6 (C^6^–Ura), 104.3 (C^5^–Ura), 86.5 (C^2^-cod^B^), 81.9 (C^1^-cod^A^), 81.1 (C^2^-cod^A^), 78.9 (C^1^-cod^B^), 70.7 (C^5^-cod^B^), 70.1 (C^6^-cod^A^), 69.9 (C^6^-cod^B^), 69.8 (C^5^-cod^A^), 33.3 (C^7^-cod^B^), 32.1 (C^7^-cod^A^), 31.3 (C^3^-cod^B^), 31.1 (C^4^-cod^A^), 30.5 (C^8^-cod^A^), 30.9 (C^4^-cod^B^), 30.1 (C^3^-cod^A^), 29.2 (C^8^-cod^B^).
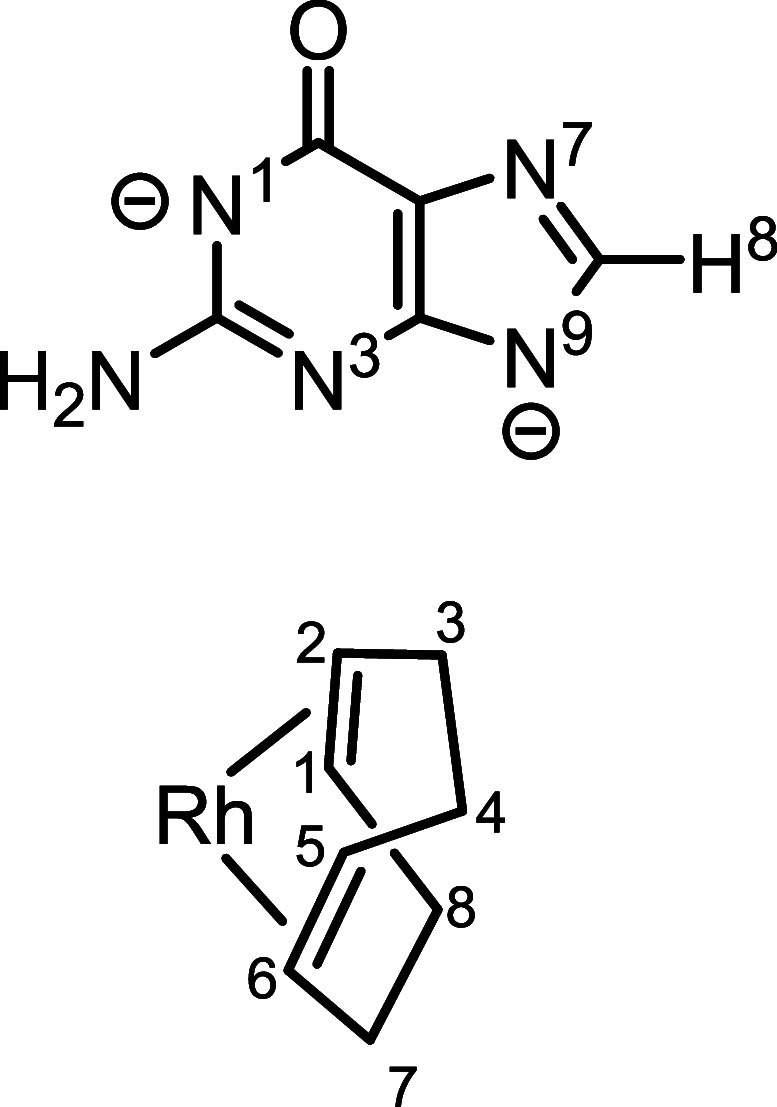


#### Synthesis of [{Rh_2_(cod)_2_(μ_4_-κ^4^ *N^1^*,*N* ^7^,*N* ^3^,*N* ^9^-Gua)}_4_] (**4**)

1.6 mL of a KOH/MeOH solution (0.47 M, 0.75 mmol) was added to guanine (49.9 mg, 0.33 mmol) in 3 mL of MeOH resulting in a white suspension. To this suspension [{Rh­(μ-Cl)­(cod)}_2_] (152.9 mg, 0.31 mmol) was added and refluxed for 15 min. The resulting yellow suspension was cooled to room temperature, the volatiles were removed under vacuum and the residue was extracted into CH_2_Cl_2_ (3 × 10 mL). The resulting orange solution was evaporated *in vacuo* to 1 mL, and 4 mL of hexane were added, yielding an orange solid which was collected by filtration, washed and vacuum-dried. Yield: 89.3 mg (50%). Yellow crystals suitable for single-crystal X-ray diffraction were grown from slow evaporation of a CHCl_3_ solution at 25 °C. IR v/cm^–1^ (ATR): 1612 (s), 1514 (s), 1465 (s), 1403 (s), 1174 (w), 1072 (w). ^1^H NMR (500 MHz, CDCl_3_, 25 °C): δ = 7.83 (s, 4H, H^8^-Gua), 7.71 (s, 8H, NH_2_-Gua), 4.83 (t, *J* = 6.4 Hz, 4H, H^1^-cod^A^), 4.42 (m, 8H, H^1^-cod^B^ + H^5^-cod^B^), 4.23 (m, 8H, H^5^-cod^A^ + H^2^-cod^A^), 4.14 (dd, *J* = 4.8 Hz, 4H, H^6^-cod^A^), 4.11 (m, 4H, H^6^-cod^B^), 3.45 (m, 4H, H^2^-cod^B^), 3.21 (ddd, *J* = 17.5, 14.8, 7.4 Hz, 4H, H^8a^-cod^A^), 2.97 (m, 4H, H^4a^-cod^A^), 2.78 (m, 12H, H^8a^-cod^B^ + H^4a^-cod^B^ + H^3a^-cod^A^), 2.66 (m, 4H, H^7b^-cod^A^), 2.50 (m, 8H, H^3a^-cod^B^ + H^7b^-cod^B^), 2.23 (m, 4H, H^8b^-cod^A^), 2.10 (m, 4H, H^4b^-cod^A^), 1.95 (m, 4H, H^3b^-cod^A^), 1.84 (m, 12H, H^7b^-cod^A^ + H^8b^-cod^B^ + H^4b^-cod^B^), 1.67 (m, 8H, H^3b^-cod^B^ + H^7b^-cod^B^). ^13^C­{^1^H} NMR (125.7 MHz, CDCl_3_, 25 °C): δ = 162.7 (OC6-Gua), 157.2 (C^2+4^-Gua), 142.5 (C^8^-Gua), 118.5 (C^5^-Gua), 88.1 (C^1^-cod^A^), 87.6 (C^6^-cod^A^), 82.8 (C^5^-cod^B^), 82.4 (C^6^-cod^B^), 81.7 (C^5^-cod^A^), 78.9 (C^1^-cod^B^), 75.3 (C^2^-cod^B^), 74.9 (C^2^-cod^A^), 33.0 (C^8^-cod^B^), 32.7 (C^8^-cod^A^), 31.8 (C^4^-cod^A^), 31.3 (C^3^-cod^A^), 31.3 (C^4^-cod^B^), 30.2 (C^3^-cod^B^), 30.0 (C^7^-cod^B^), 29.8 (C^7^-cod^A^). HR-MALDI-TOF^+^: *m*/*z* (%) = calcd for [Rh_8_C_84_H_108_N_20_O_4_] [M]^+^ 2284.1297, found: 2284.1225 (err [mDa] = 7.2). Anal. Calcd for C_84_H_108_O_4_N_20_Rh_8_·CH_2_Cl_2_: C 43.08 H 4.68, N 11.82; found: C 43.02, H 5.30, N 10.76.

In samples prepared and handled under inert conditions, **4**:**5** ratios of ca. 2.2:1 were observed by ^1^H NMR spectroscopy. Selected ^1^H NMR signals for **5** (500 MHz, CDCl_3_, 25 °C): δ = 8.52 (s, 8H, NH_2_-Gua), 7.50 (s, 4H, H^8^-Gua), 4.70 (m, 8H, cod), 4.61 (m, 8H, cod), 3.96 (vt, *J* = 7.2 Hz, 4H, cod), 3.83 (vt, *J* = 6.1 Hz, 8H, cod).

## Supplementary Material



## Notes

Experimental procedures; additional NMR spectra; DOSY NMR data; electrochemical data; hydrogen bonding parameters; crystallographic data (PDF)
