# Supplementary material for: Low-Valent Rhodium and Iridium Assemblies Directed by Uracilate and Guaninate Linkers
Source: Inorg Chem. 2026 Jul 6;65(28):16190–8. doi: 10.1021/acs.inorgchem.6c01485 (PMC13390032; doi:10.1021/acs.inorgchem.6c01485)
Supplement: Supplementary file 1 [file ic6c01485_si_001.pdf]

Low-Valent Rhodium and Iridium Assemblies Directed by Uracilate and Guaninate Linkers

Adrián Badía, Laura Asensio, M. Pilar del Río, José A. López, B. Eva Villarroya, Ana M. Geer, Cristina Tejel**

Instituto de Síntesis Química y Catálisis Homogénea (ISQCH), Departamento de Química Inorgánica, CSIC–Universidad de Zaragoza, Pedro Cerbuna 12, 50009 Zaragoza, Spain

Email: anageer@unizar.es; ctejel@unizar.es

Table of Contents

NMR spectra 2

Supplementary DOSY NMR Data 10

Electrochemistry 11

X-Ray Crystallographic Details 12

References 15

NMR spectra

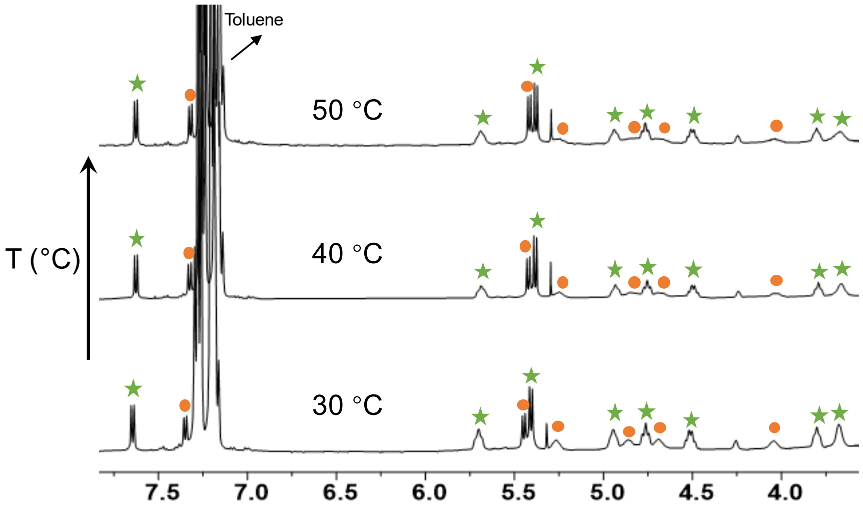

Relative distribution from
^{1}H NMR integration

T (°C)	1 (%)	3 (%)
50	57.7	42.3
40	64.5	35.5
30	69.3	30.7

Figure S1. Variable temperature ^{1}H NMR monitoring of the equilibrium between [{Rh$_2$(cod)$_2$(μ$_4$-κ^2*N*^{1},*N*^{3}:κ^2*O*^{2},*O*^{4}-Ura)}$_3$] (**1**) and [{Rh$_2$(cod)$_2$(Ura)}$_4$] (**3**) in CDCl$_3$ at 30, 40, 50 ºC, using toluene as an internal standard. The table on the right summarizes the approximate relative distribution of **1** and **3** determined from the integration of selected resonances.

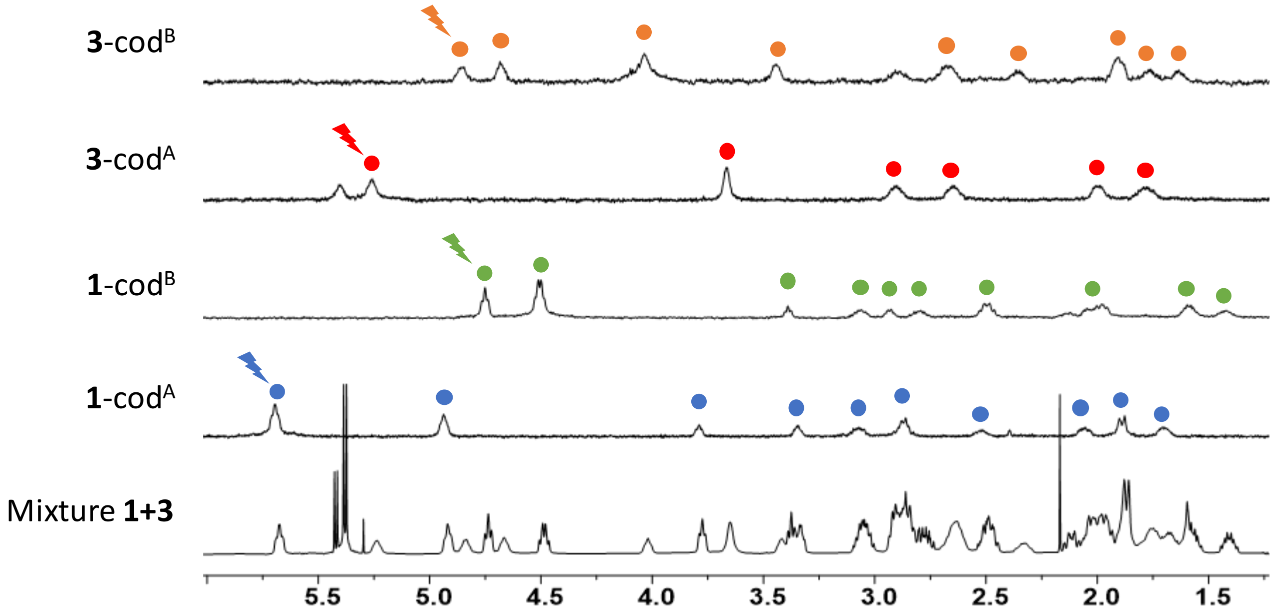

Figure S2. Selected regions of the ^{1}H NMR spectrum (bottom) and ^{1}H-selTOCSY under irradiation of the indicated signals for the equilibrated solution of [{Rh$_2$(cod)$_2$(μ$_4$-κ^2*N*^{1},*N*^{3}:κ^2*O*^{2},*O*^{4}-Ura)}$_3$] (**1**) and [{Rh$_2$(cod)$_2$(Ura)}$_4$] (**3**) in CDCl$_3$ at 25 ºC.

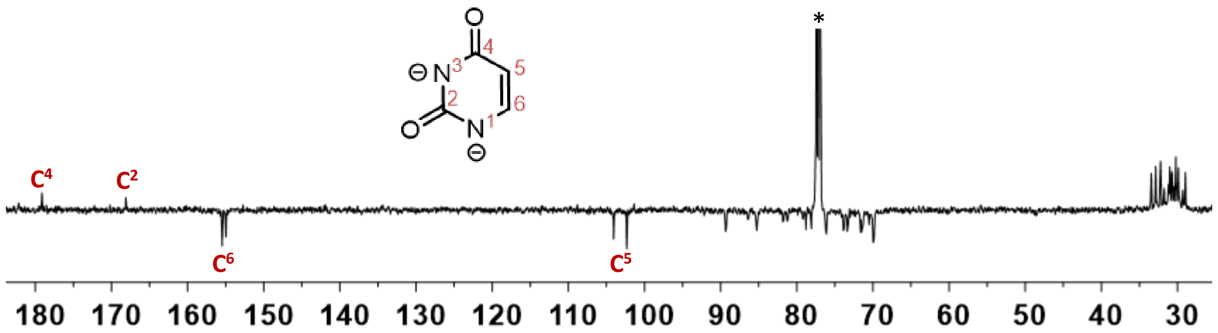

Figure S3. ^{13}C{^{1}H}-APT NMR spectrum of the equilibrated solution of [{$Rh_2(cod)_2(\mu_4-\kappa^2N^1,N^3:\kappa^2O^2,O^4$-Ura)}$_3$] (**1**) and [{$Rh_2(cod)_2$(Ura)}$_4$] (**3**) in $CDCl_3$ at 25 ºC. The asterisk (*) indicates the residual solvent signal.

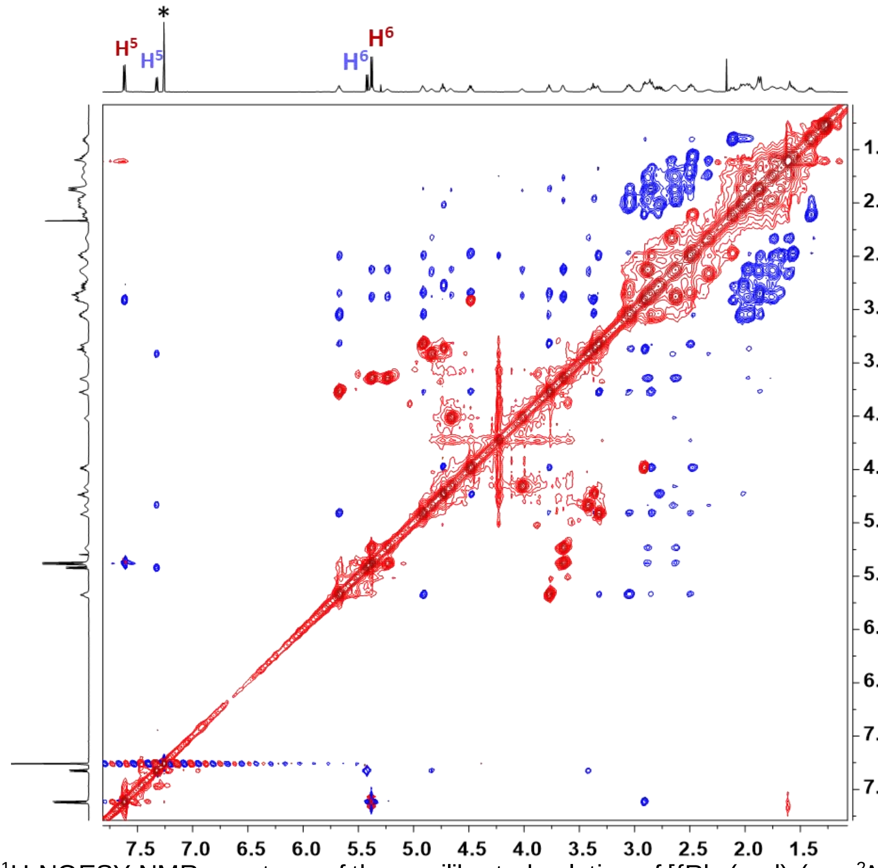

Figure S4. ^{1}H,^{1}H-NOESY NMR spectrum of the equilibrated solution of [{$Rh_2(cod)_2(\mu_4-\kappa^2N^1,N^3:\kappa^2O^2,O^4$-Ura)}$_3$] (**1**) and [{$Rh_2(cod)_2$(Ura)}$_4$] (**3**) in $CDCl_3$ at 25 ºC. The asterisk (*) indicates the residual solvent signal.

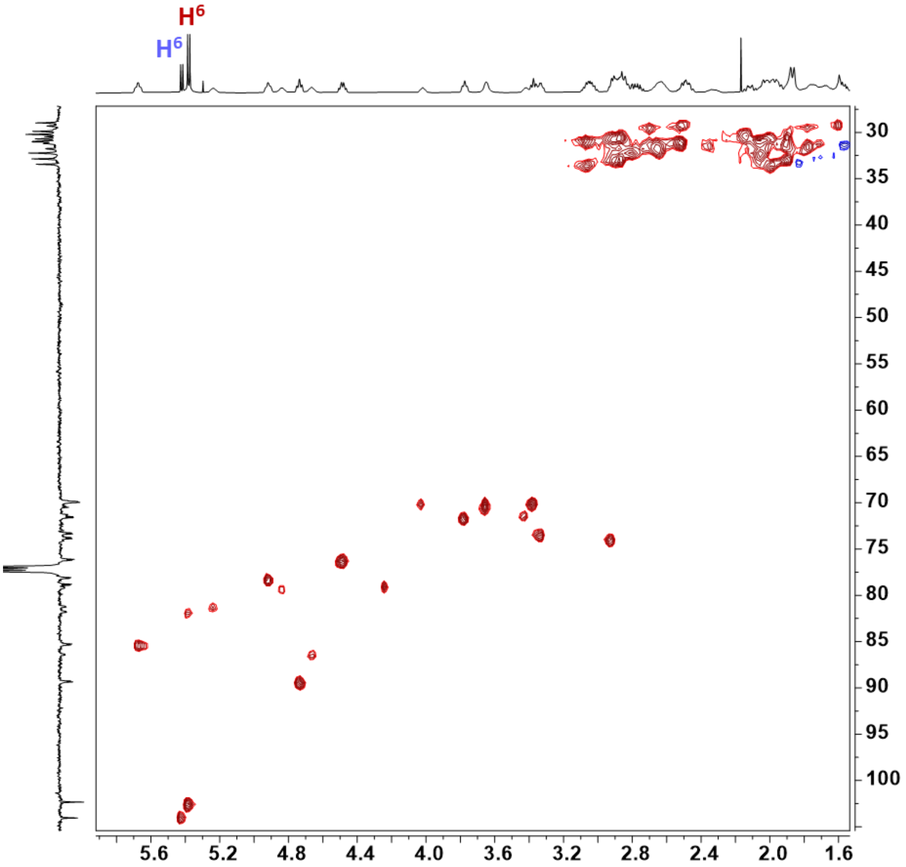

Figure S5. Selected region of $^{1}H,^{13}C$-HSQC NMR spectrum of the equilibrated solution of [{$Rh_2(cod)_2(\mu_4$-$\kappa^2 N^1,N^3{:}\kappa^2 O^2,O^4$-Ura)}$_3$] (**1**) and [{$Rh_2(cod)_2$(Ura)}$_4$] (**3**) in $CDCl_3$ at 25 ºC.

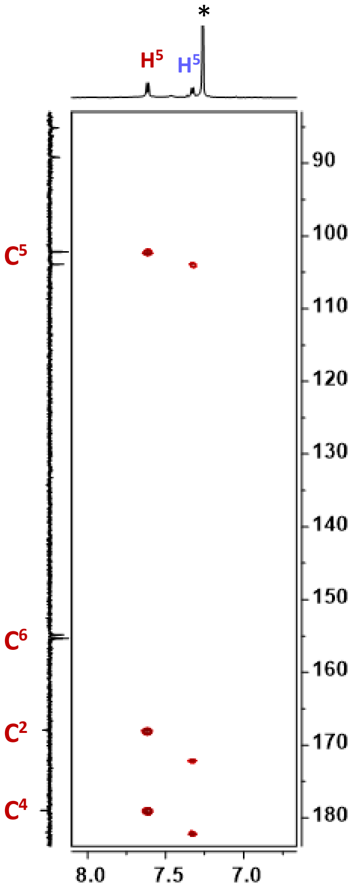

Figure S6. Selected region of the $^{1}H,^{13}C$-HMBC NMR spectrum of the equilibrated solution of [{$Rh_2(cod)_2(\mu_4$-$\kappa^2N^1,N^3:\kappa^2O^2,O^4$-Ura)}$_3$] (**1**) and [{$Rh_2(cod)_2$(Ura)}$_4$] (**3**) in $CDCl_3$ at 25 ºC. The asterisk (*) indicates the residual solvent signal.

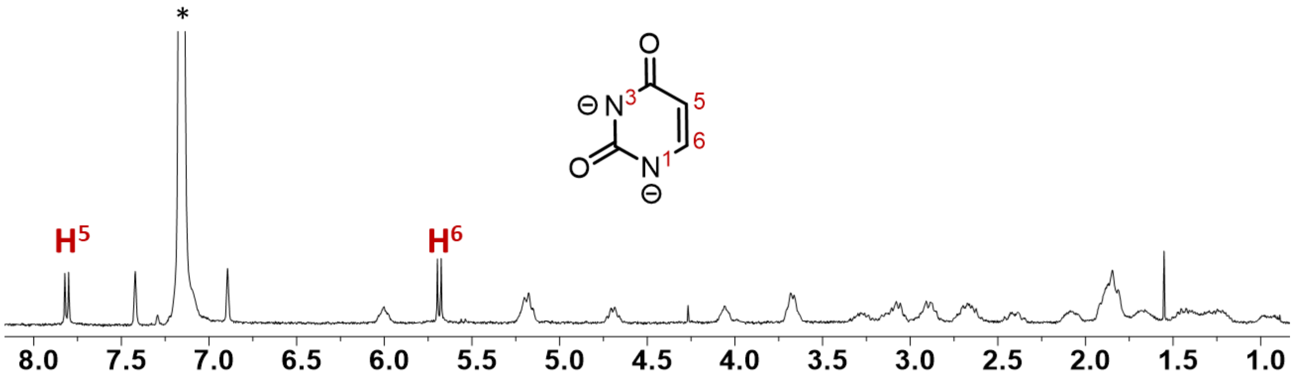

Figure S7. ^{1}H NMR spectrum of [{$Rh_2(cod)_2(\mu$-Ura)}$_4$] (**3**) in C_6D_6. The asterisk (*) indicates the residual solvent signal.

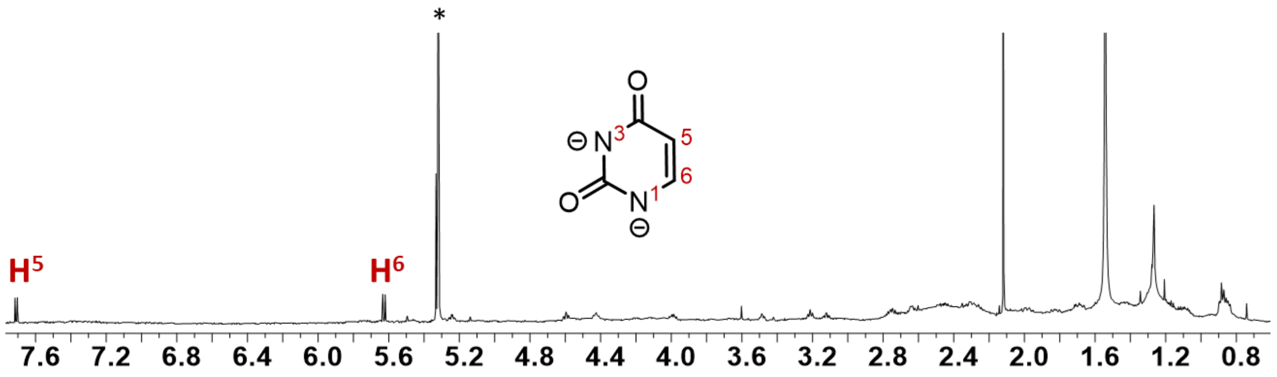

Figure S8. ^{1}H NMR spectrum of [{Ir$_2$(cod)$_2$(μ$_4$-κ^2N^1,N^3:κ^2O^2,O^4-Ura)}$_3$] (**2**) in CD_2Cl_2. The asterisk (*) indicates the residual solvent signal.

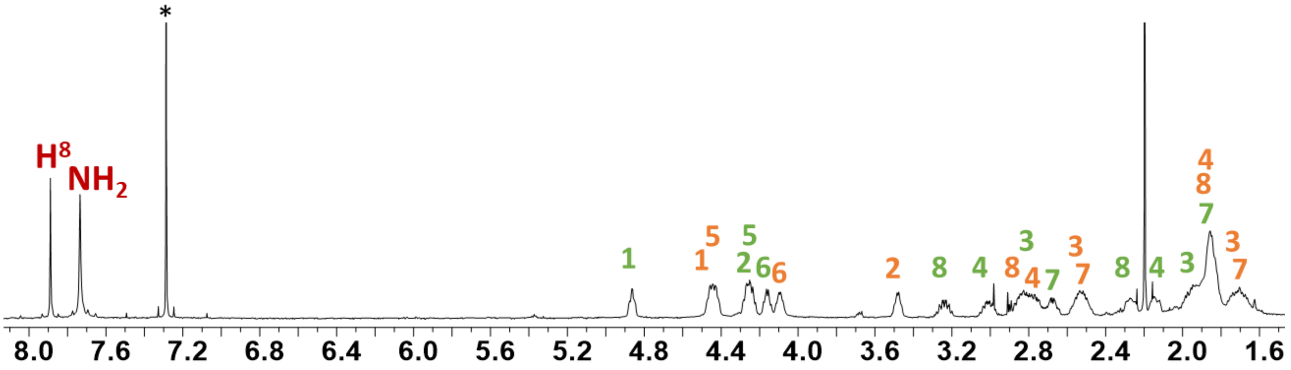

Figure S9. ^{1}H NMR spectrum of [{Rh$_2$(cod)$_2$(μ$_4$-κ^4N^1,N^7,N^3,N^9-Gua)}$_4$] (**4**) in $CDCl_3$. The asterisk (*) indicates the residual solvent signal.

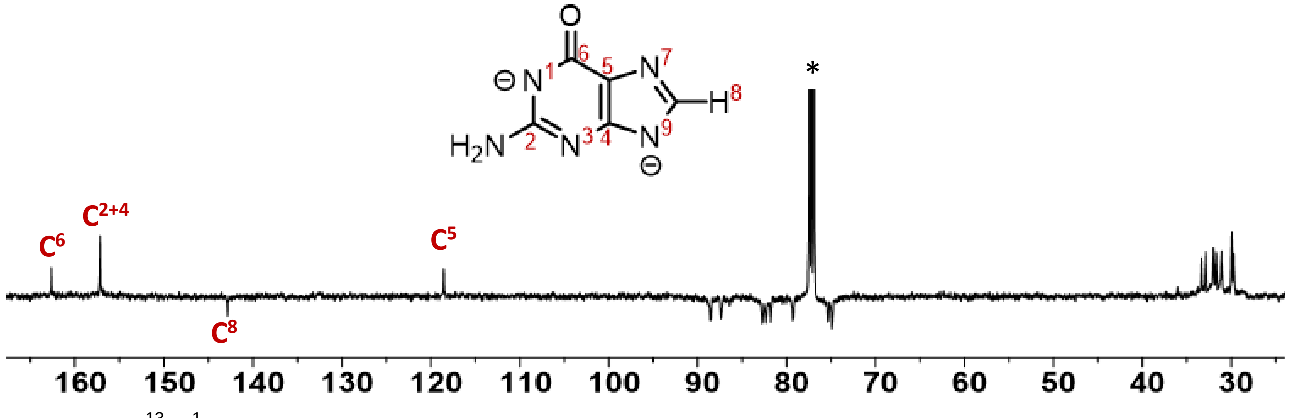

Figure S10. ^{13}C{^{1}H}-APT NMR spectrum of [{Rh$_2$(cod)$_2$(μ$_4$-κ^4N^1,N^7,N^3,N^9-Gua)}$_4$] (**4**) in $CDCl_3$. The asterisk (*) indicates the residual solvent signal.

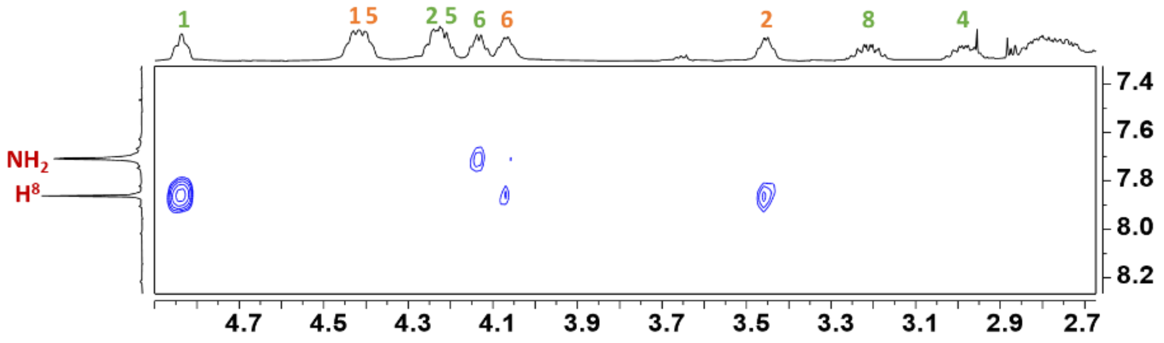

Figure S11. Selected region of the ^{1}H,^{1}H-NOESY NMR spectrum of [{$Rh_2(cod)_2(\mu_4\text{-}\kappa^4 N^1,N^7,N^3,N^9\text{-Gua})$}$_4$] (**4**) in $CDCl_3$.

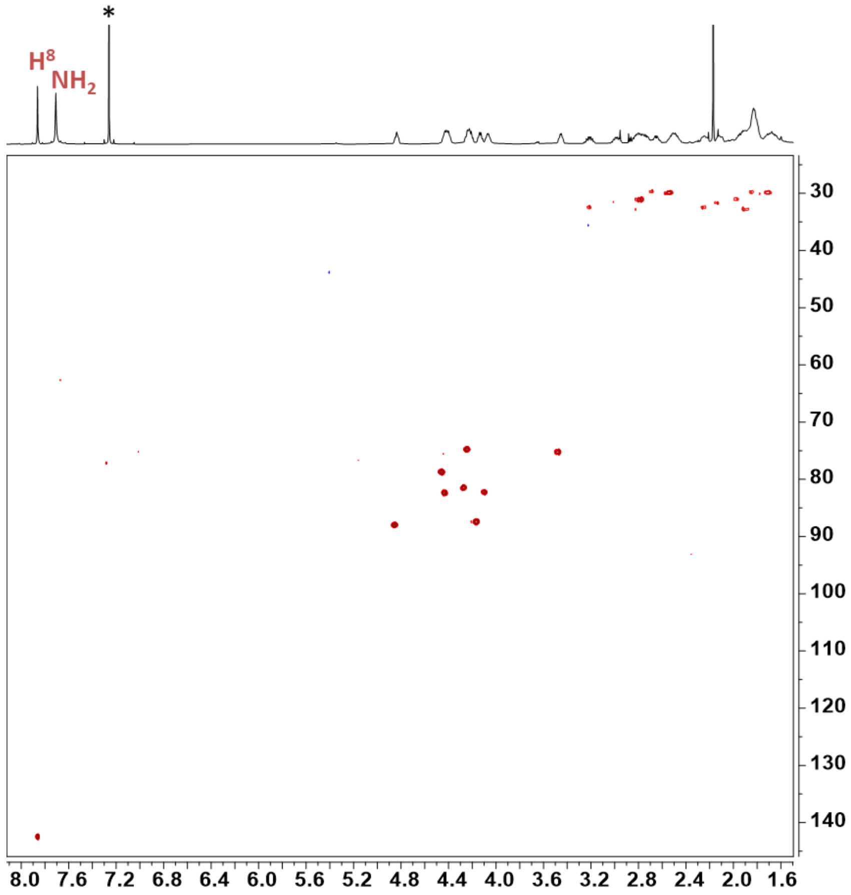

Figure S12. ^{1}H,^{13}C-HSQC NMR spectrum of [{$Rh_2(cod)_2(\mu_4\text{-}\kappa^4 N^1,N^7,N^3,N^9\text{-Gua})$}$_4$] (**4**) in $CDCl_3$. The asterisk (*) indicates the residual solvent signal.

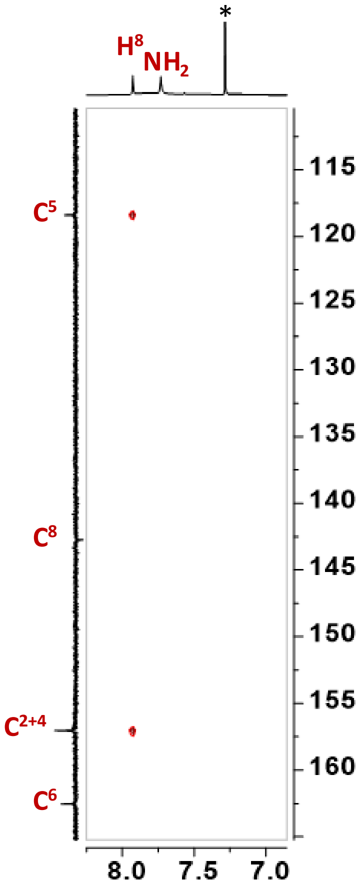

Figure S13. Selected region of the $^{1}H,^{13}C$-HMBC NMR spectrum of [{$Rh_2(cod)_2(\mu_4\text{-}\kappa^4 N^1,N^7,N^3,N^9$-Gua)}$_4$] (**4**) in $CDCl_3$. The asterisk (*) indicates the residual solvent signal.

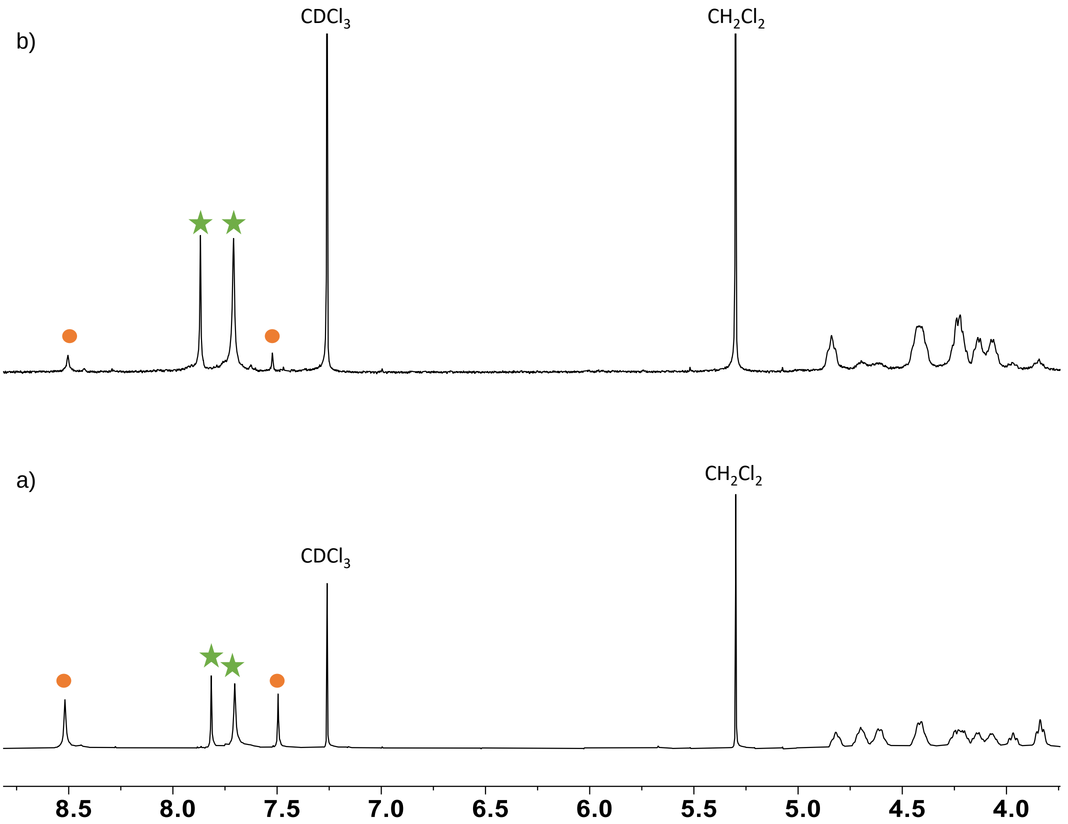

Figure S14. ^{1}H NMR spectra ($CDCl_3$, 25 ºC) of the yellow solid isolated after refluxing the reaction mixture for 1 h: (a) after immediate workup; (b) after stirring at 25 °C for 16 h before workup. Guanine signals corresponding to complex **4** are marked with green stars, while those corresponding to complex **5** are marked with orange circles.

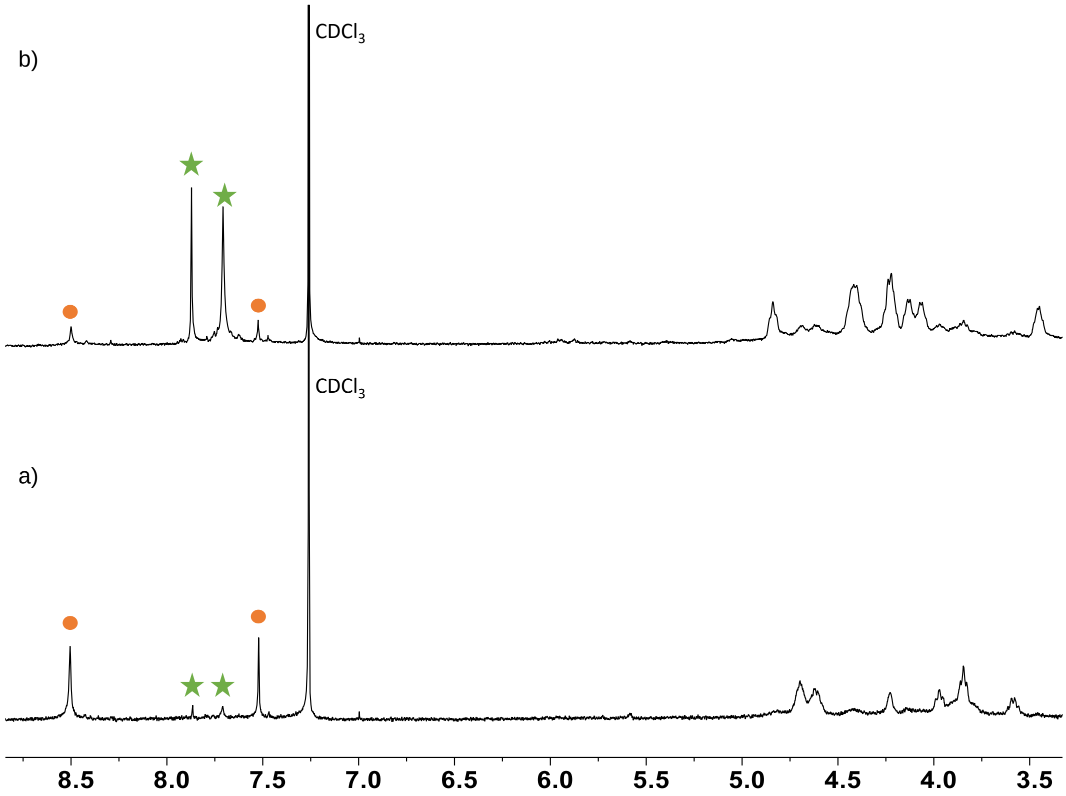

Figure S15. ^{1}H NMR monitoring of the evolution in solution of [{Rh_2(cod)$_2$(Gua)}$_4$] (**5**) to [{Rh_2(cod)$_2$(μ_4-$\kappa^4$$N^1$,$N^7$,$N^3$,$N^9$-Gua)}$_4$] (**4**) in $CDCl_3$ at 25 ºC. (a) after 10 min and (b) after 20 h. The asterisk (*) indicates the residual solvent signal. Guanine signals corresponding to complex **4** are marked with green stars, while those corresponding to complex **5** are marked with orange circles.

Supplementary DOSY NMR Data

Table S1. Summary of ^{1}H DOSY NMR results.

Species	Diffusion coefficients (D, $m^2 s^{-1}$)	Hydrodynamic radii (r_H, Å)
[{$Rh_2(cod)_2(\mu_4\text{-}\kappa^2 N^1,N^3{:}\kappa^2 O^2,O^4$-Ura)}$_3$] (**1**)	6.35×10^{-10}	6.74
[{$Rh_2(cod)_2$(Ura)}$_4$] (**3**)	5.06×10^{-10}	8.46
[{$Rh_2(cod)_2(\mu_4\text{-}\kappa^4 N^1,N^7,N^3,N^9$-Gua)}$_4$] (**4**)	4.72×10^{-10}	9.22
[{$Rh_2(cod)_2$(Gua)}$_4$] (**5**)	4.64×10^{-10}	9.38

Molecular model of 3:

Based on the crystallographic structure of [{$Rh_2(cod)_2(\mu_4\text{-}\kappa^2 N^1,N^3{:}\kappa^2 O^2,O^4$-Ura)}$_3$] (**1**), a molecular model for the higher nuclearity species [{$Rh_2(cod)_2$(Ura)}$_4$] (**3**) was generated using BIOVIA Materials Studio. The model was constructed by expanding the hexanuclear framework through the incorporation of two additional rhodium atoms and one additional uracilate ligand. The coordination spheres of the added Rh centers were completed with cod ligands in order to preserve the square planar Rh(I) environment observed experimentally. The resulting structure was then geometrically optimized using the universal force field implemented in the Forcite module of Materials Studio.

This model shows that an octanuclear uracilate based Rh(I) assembly with a larger molecular volume is geometrically feasible, in agreement with the larger hydrodynamic radius determined for **3** by DOSY NMR spectroscopy.

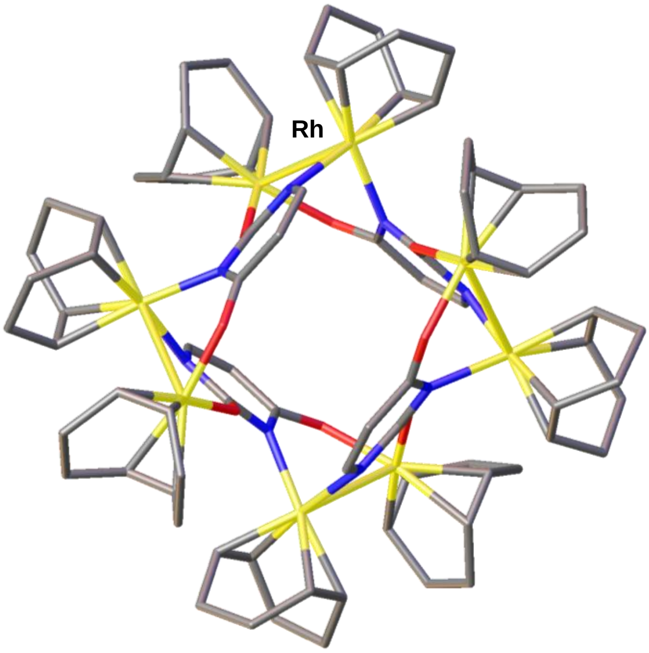

Figure S16. Molecular model of [{$Rh_2(cod)_2$(Ura)}$_4$] (**3**).

Electrochemistry

Cyclic voltammetry measurements of **1** and **4** were collected under Ar using a WaveNowWireless potentiostat (Pine Research Instrumentation) in an undivided three electrode cell, with a glassy carbon working electrode (3 mm diameter), a platinum wire counter electrode, and a silver wire pseudoreference electrode. Glassy carbon electrodes were polished using a Milli-Q water slurry of 0.05 μm alumina powder, rinsed and sonicated in Milli-Q water, and finally rinsed with acetone. A 0.1 M solution of tetrabutylammonium hexafluorophosphate ($[NBu_4][PF_6]$) in CH_2Cl_2 was used as the supporting electrolyte. All data were referenced to an internal ferrocene standard (ferrocenium/ferrocene reduction potential under stated conditions).

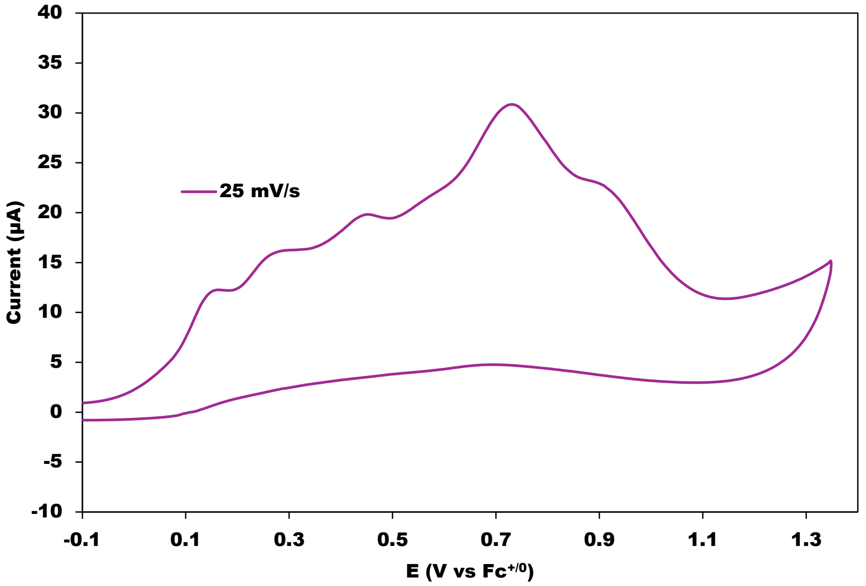

Figure S17. Cyclic voltammogram of **4** (1.0 mM) in CH_2Cl_2 with 0.1 M $[NBu_4][PF_6]$ as a supporting electrolyte recorded at 25 mV/s.

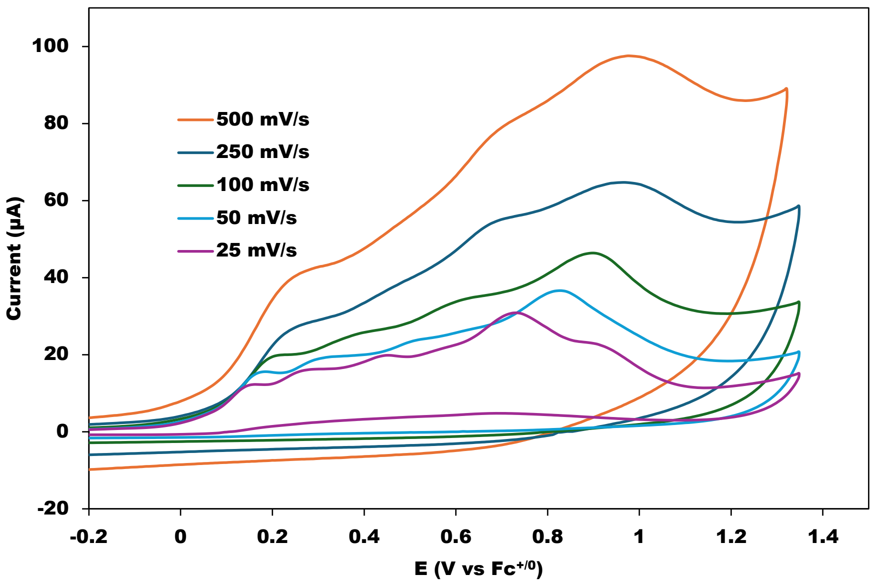

Figure S18. Cyclic voltammogram of **4** (1.0 mM) in CH_2Cl_2 with 0.1 M $[NBu_4][PF_6]$ as a supporting electrolyte recorded at variable scan rates (25 mV/s to 500 mV/s).

X-Ray Crystallographic Details

Intensity measurements were collected with a Bruker Smart Apex (**1**, **2**) or Apex-II (**4**) diffractometers with graphite-monochromated $Mo_{K\alpha}$ radiation. Single crystals were coated with a protecting perfluoropolyether oil in the mother liquor, mounted on a fiber, and cooled to 100(2) K with an open-flow nitrogen gas. Data were collected using ω-scans of 0.3º; a semi-empirical absorption correction was applied to the data sets with the multi-scan[1] method. The structures were solved by direct methods with SHELXS-2013[2] and refined by full-matrix least-squares on F^2 with the program SHELXL-2016,[3] in the WINGX[4] package. All non-hydrogen atoms were refined with anisotropic displacement parameters, except three disordered trichloromethane molecules in (**4**) that were refined with several common isotropic displacement parameters. The hydrogen atoms were located in the difference-Fourier maps or geometrically calculated, and refined by the riding mode, including the isotropic displacement parameters. The three characterizations include modelled solvent molecules (three CH_2Cl_2 molecules in (**1**) and (**2**) and six $CHCl_3$ molecules in (**4**)), but also the SQUEEZE[5] procedure was used to account for other severe disordered solvent molecules, three dichloromethane molecules per unit cell in (**1**) and (**2**), and four chloroform molecules per unit cell in (**4**); they have been taken into account in the chemical formula, F000 and density. These numbers of solvent molecules were inferred from the solvent accessible volume per unit cell (552 $Å^{-3}$ in (**1**), 534 $Å^{-3}$ in (**2**) and 703 $Å^{-3}$ in (**4**)) and the number of electrons in these voids (124 e^-/unit cell in (**1**), 137 e^-/unit cell in (**2**) and 231 e^-/unit cell in (**4**)), as calculated by the SQUEEZE program.

Selected crystallographic data for [{$Rh_2(cod)_2(\mu_4$-κ^2*N*^{1},*N*^{3}:κ^2*O*^{2},*O*^{4}-Ura)}$_3$]·4.5CH_2Cl_2 ([1]·4.5CH_2Cl_2). Local name ctb32ms_sq. Crystal data: $C_{64.5}H_{87}Cl_9N_6O_6Rh_6$, *M*r = 1978.91, triclinic, space group P-1, *a* = 15.8903(19), *b* = 16.034(2), *c* = 17.927(2) Å, *α* = 88.334(2), *β* = 69.353(2), *γ* = 60.570(2)º, *V* = 3664.1(8) $Å^3$, *Z* = 2, ρ_{calcd} = 1.794 g cm^{-3}, *F*(000) = 1974, *T* = 100(2) K, $Mo_{K\alpha}$ radiation (λ = 0.71073 Å, μ = 1.700 mm^{-1}). Data were collected with a red irregular block (0.080 × 0.065 × 0.060 mm). Of 30866 measured reflections (2θ: 3.2-52.0º), 14310 were unique (R_{int} = 0.0482). Final agreement factors were R_1 = 0.0506 (11129 observed reflections) and wR_2 = 0.1161. Data/restraints/parameters 14310/54/784; GOF = 1.061. Largest peak and hole in the final difference map 1.609 and -1.214 $e^-/Å^{-3}$.

Selected crystallographic data for [{$Ir_2(cod)_2(\mu_4$-κ^2*N*^{1},*N*^{3}:κ^2*O*^{2},*O*^{4}-Ura)}$_3$]·4.5CH_2Cl_2 ([2]·4.5CH_2Cl_2). Local name ctb177ms_sq. Crystal data: $C_{64.5}H_{87}Cl_9Ir_6N_6O_6$, *M*r = 2514.65, triclinic, space group P-1, *a* = 15.9873(14), *b* = 16.0749(14), *c* = 17.9577(16) Å, *α* = 88.1184(11), *β* = 69.5559(10), *γ* = 60.3254(9)º, *V* = 3701.2(6) $Å^3$, *Z* = 2, ρ_{calcd} = 2.256 g cm^{-3}, *F*(000) = 2358, *T* = 100(2) K, $Mo_{K\alpha}$ radiation (λ = 0.71073 Å, μ = 11.121 mm^{-1}). Data were collected with a red irregular block (0.20 × 0.14 × 0.09 mm). Of 30392 measured reflections (2θ: 3.0-52.0º), 14383 were unique (R_{int} = 0.0291). Final agreement factors were R_1 = 0.0470 (13196 observed reflections) and wR_2 = 0.1106. Data/restraints/parameters 14383/48/784; GOF = 1.069. Largest peak and hole in the final difference map 2.173 and -2.650 $e^-/Å^{-3}$.

Selected crystallographic data for [{$Rh_2(cod)_2(\mu_4$-κ^4 *N*^{1},*N*^{7},*N*^{3},*N*^{9}-Gua)}$_4$]·8$CHCl_3$ ([4]·8$CHCl_3$). Local name jal29ms_sq. Crystal data: $C_{92}H_{116}Cl_{24}N_{20}O_4Rh_8$, *M*r = 3240.12, triclinic, space group P-1, *a* = 15.537(3), *b* = 15.672(3), *c* = 24.545(4) Å, *α* = 96.511(3), *β* = 101.764(3), *γ* = 93.565(3)º, *V* 5790.8(18) $Å^3$, *Z* = 2, ρ_{calcd} = 1.858 g cm^{-3}, *F*(000) = 3216, *T* = 100(2) K, $Mo_{K\alpha}$ radiation (λ = 0.71073 Å, μ = 1.721 mm^{-1}). Data were collected with an orange irregular block (0.14 × 0.12 × 0.03 mm). Of 53215 measured reflections (2θ: 1.7-52.0º), 22743 were unique (R_{int} = 0.0952). Final agreement factors were R_1 = 0.0866 (12757 observed reflections) and wR_2 = 0.2145. Data/restraints/parameters 22743/60/1230; GOF = 1.043. Largest peak and hole in the final difference map 2.599 and -1.795 $e^-/Å^{-3}$.

Crystallographic data for **([1]·4.5CH_2Cl_2)** (CCDC 2537351), **([2]·4.5CH_2Cl_2)** (CCDC 2537352) and **([4]·8$CHCl_3$)** (CCDC 2537353) have been submitted to the CCDC.

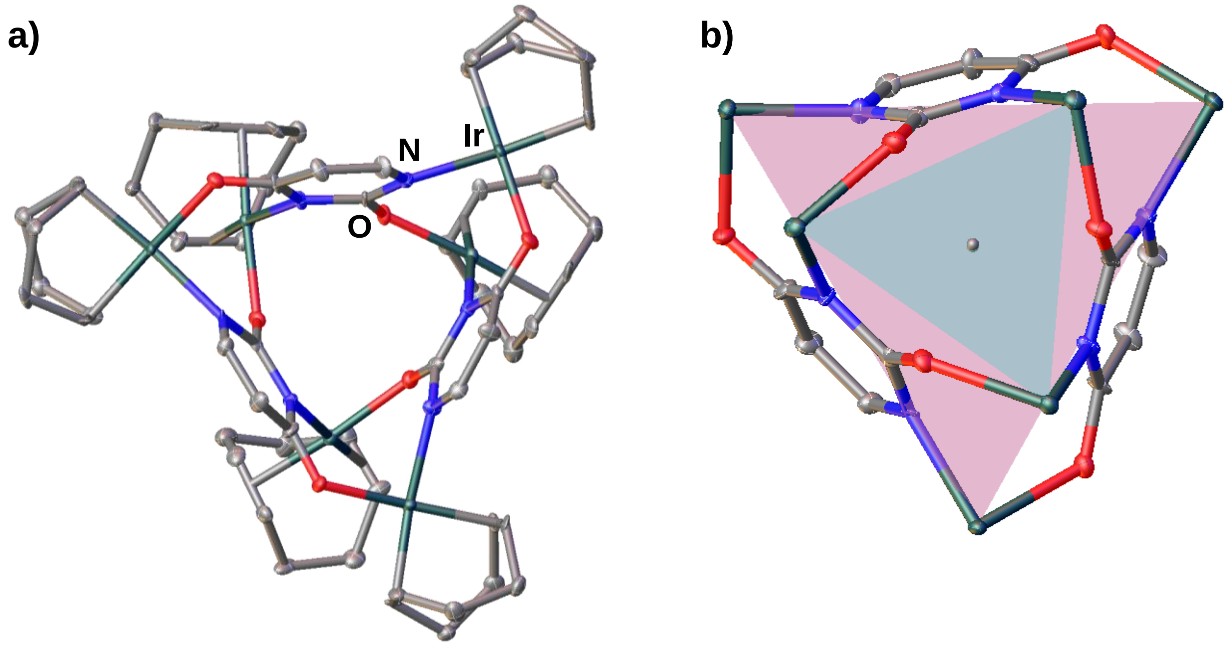

Figure S19. (a) Molecular structure of hexanuclear $[\{Ir_2(cod)_2(\mu_4\text{-}\kappa^2 N^1,N^3\text{:}\kappa^2 O^2,O^4\text{-Ura})\}_3]$ (**2**) (b) Core representation of **2** highlighting the two triangular Ir_3 planes. Hydrogen atoms and solvent molecules are omitted for clarity.

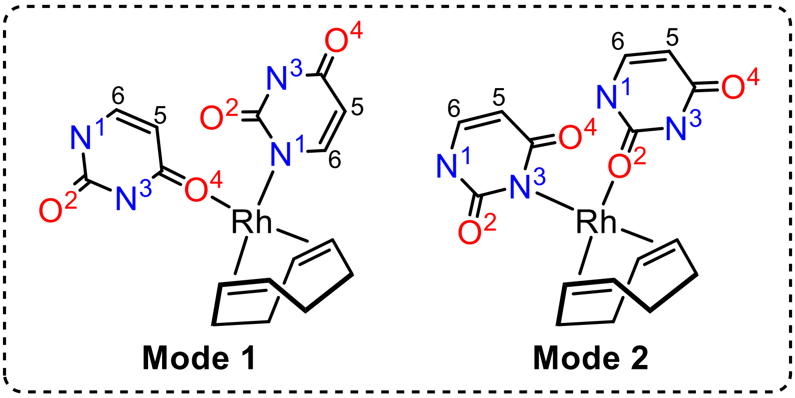

Figure S20. Illustration of the two coordination modes around the Rh centers in $[\{Rh_2(cod)_2(\mu_4\text{-}\kappa^2 N^1,N^3\text{:}\kappa^2 O^2,O^4\text{-Ura})\}_3]$ (**1**), bonded to N1/O4 or N3/O2, with atom numbering indicated.

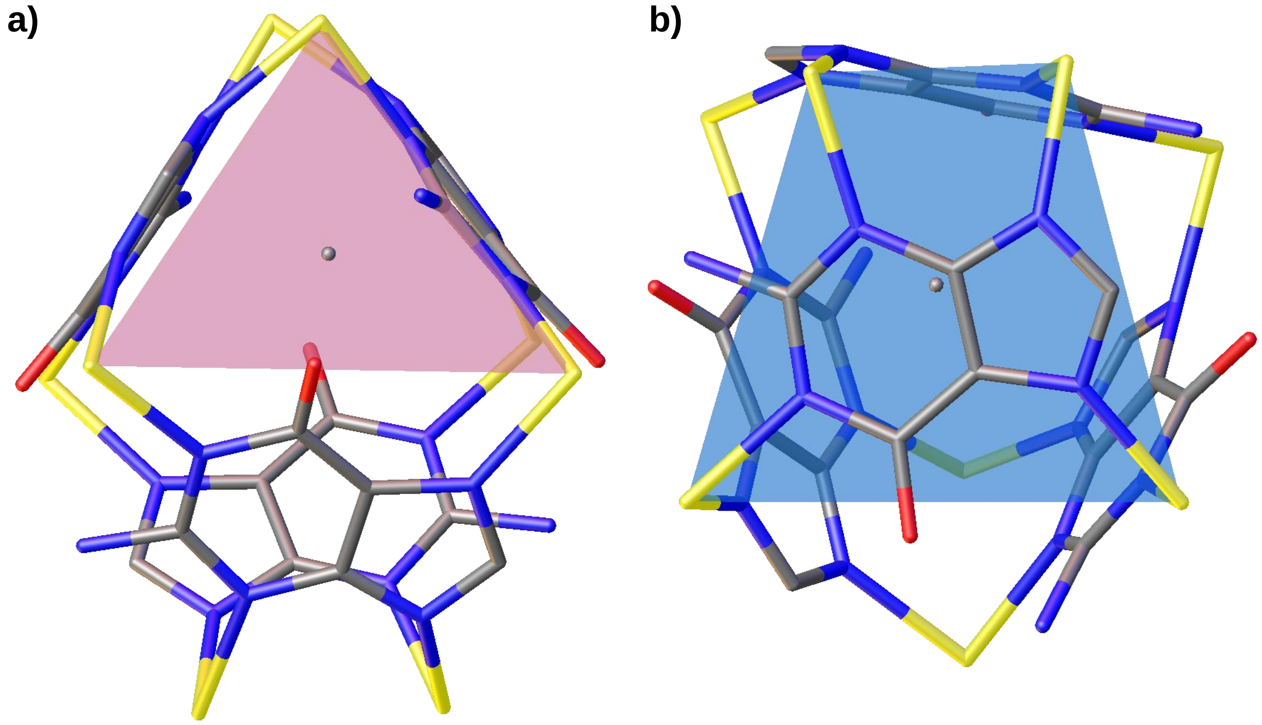

Figure S21. Core representation of $[\{Rh_2(cod)_2(\mu_4\text{-}\kappa^4 N^1,N^7,N^3,N^9\text{-Gua})\}_4]$ (**4**), highlighting (a) a triangular Rh_3 face (b) a trapezoidal Rh_4 face. Hydrogen atoms, solvent molecules and cod ligands are omitted for clarity.

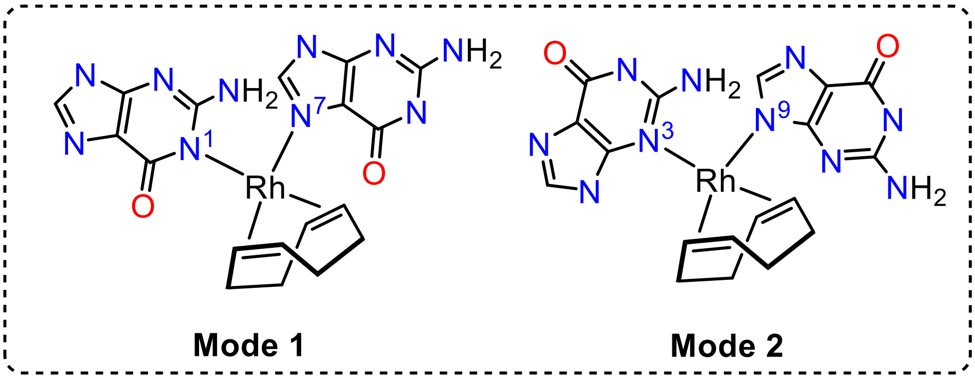

Figure S22. Illustration of the two coordination modes around the Rh centers in $[\{Rh_2(cod)_2(\mu_4\text{-}\kappa^4 N^1,N^7,N^3,N^9\text{-Gua})\}_4]$ (**4**), bonded to N1/N7 or N3/N9, with atom numbering indicated.

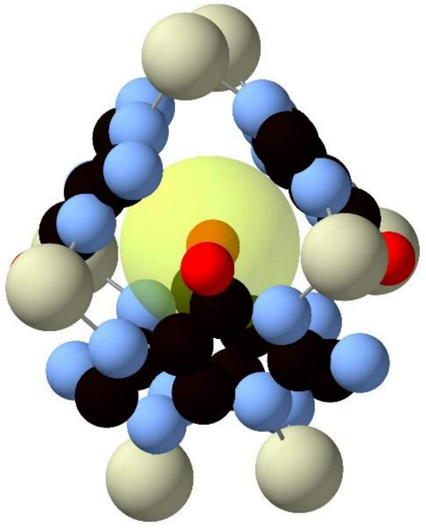

Figure S23. Representation of the internal cavity of [{Rh_2(cod)$_2$(μ_4-κ^4*N*^{1},*N*^{7},*N*^{3},*N*^{9}-Gua)}$_4$] (**4**), illustrated by the inclusion of a 5 Å diameter sphere. Hydrogen atoms, solvent molecules and cod ligands are omitted for clarity.

Table S2. Selected hydrogen-bond parameters for complex [{Rh_2(cod)$_2$(μ_4-κ^4*N*^{1},*N*^{7},*N*^{3},*N*^{9}-Gua)}$_4$] (**4**).

D–H···A	H···A (Å)	D···A (Å)	∠DHA (°)
**N5–H5B···O3**	2.10	2.799(12)	140.6
**N10–H10B···O4**	1.87	2.845(14)	170.5
**N15–H15A···O2**	2.14	2.850(14)	126.3
**N20–H20B···O1**	2.01	2.826(13)	144.4
**C108–H108···O2 ($CHCl_3$)**	2.01	2.99(3)	169.4
**C109–H109···O2 ($CHCl_3$)**	2.09	3.08(4)	170.9

References

(1) Sheldrick, G.M. SADABS, Program for Bruker Area Detector Absorption Correction. Bruker AXS, Madison, WI (USA), 1997.

(2) Sheldrick, G. M. A Short History of SHELX. *Acta Crystallogr. A* **2008**, *64* (1), 112–122. https://doi.org/10.1107/S0108767307043930.

(3) Sheldrick, G. M. Crystal Structure Refinement with *SHELXL*. *Acta Crystallogr. Sect. C Struct. Chem.* **2015**, *71* (1), 3–8. https://doi.org/10.1107/s2053229614024218.

(4) Farrugia, L. J. *WinGX* and *ORTEP for Windows*: An Update. *J. Appl. Crystallogr.* **2012**, *45* (4), 849–854. https://doi.org/10.1107/s0021889812029111.

(5) Spek, A. L. PLATON SQUEEZE: A Tool for the Calculation of the Disordered Solvent Contribution to the Calculated Structure Factors. *Acta Crystallogr. Sect. C Struct. Chem.* **2015**, *71* (1), 9–18. https://doi.org/10.1107/S2053229614024929.
